# Localization and ambiguity resolution algorithm for time-difference fusion of three satellites based on observation filtering

**DOI:** 10.1038/s41598-025-10236-2

**Published:** 2025-07-18

**Authors:** Yanli Zhang, Haoquan Wang, Jingfeng Zheng, Lei Hua, Rui Zhang, Jian Xie

**Affiliations:** 1 School of Information Innovation and Big Data, Shanxi Jinzhong Institute of Technology, Jinzhong, 030600 China; 2 School of Information and Communication Engineering, North China University, Taiyuan, 030051 China; 3 Electronic Information College, Northwestern Polytechnical University, Xi’an, 710072 China; 4 Xi’an Institute of Space Radio Technology, Xi’an, 710100 China; 5 Innovation Academy for Microsatellites, Chinese Academy of Sciences, Shanghai, 201304 China

**Keywords:** Passive localization, Time-difference fusion, Ambiguity resolution, Gaussian-Newton iteration, Gaussian mixture model, Capacitive Kalman filtering, Aerospace engineering, Electrical and electronic engineering, Computer science

## Abstract

To address the issues of low accuracy and time-difference ambiguity in the localization and tracking of multiple satellites, we have conducted a thorough study of the localization algorithm and the ambiguity resolution algorithm for the time-difference fusion of multiple satellites. The localization algorithm based on Gaussian-Newton iteration for time-difference fusion of three satellites is proposed. The time-difference equation observed during the satellite overhead is combined with the elevation observation equation to construct the cost function for the overdetermined case, and the nonlinear least squares problem is solved based on Gaussian-Newton iteration. For localization and tracking for time-difference fusion, the Kalman filtering combined with Gaussian mixture model (KFGMM) is proposed as the ambiguity resolution algorithm of time-difference for stationary targets; the capacitive Kalman filtering combined with Gaussian mixture model (CKFGMM) is proposed for cruising targets. The mathematical model of time-difference ambiguity is established, the calculation method of time-difference window and number of ambiguous time-difference is given, and the measurements of ambiguous time-difference are approximated by Gaussian mixture model. Experiments show that localization algorithm for time-difference fusion of multiple satellites outperforms other advanced localization methods and achieves the Cramér-Rao Lower Bound (CRLB) of fusion localization; with the increase of filtering time, the ambiguity resolution algorithm for time-difference fusion of multiple satellites can reach the Bayesian Cramér-Rao Lower Bound (BCRLB) and outperforms the algorithm combining with the direction finding assistance for ambiguity resolution.

## Introduction

 The localization for time-difference of multiple satellites adopts the time-difference between the arrival of the signal from the radar emitter to multiple satellites to realize the localization^1^. The localization principle is as follows: since the geometric positions of the multiple satellites are different with respect to the radar emitter, the arrival times of the signals to the multiple satellites must be different, and the multiple satellites receive the signals and transmit them to the ground signal stations^2–4^. Using the mutual correlation between the signals, multiple sets of time-difference can be obtained, and the product of the time-difference and the signal propagation speed is range difference of arrival (RDOA)^5,6^. The merits for time-difference localization of multiple satellites are that it only needs to measure the time-difference between satellites to achieve instantaneous localization, high accuracy and large coverage^7^. However, the drawback is that the geometric configuration of the multiple satellites keeps changing with constant motion^8,9^. When the multiple satellites is in a straight line at a certain instant in the high latitude, the radar emitters at certain locations will not be located.

Analyzed in terms of the number of observatories, each additional observatory equals one more time-difference equation. When the number of equations is larger than the unknown parameters, the cost function can be constructed to be solved by least squares estimation^10,11^. Under the same conditions, the more the number of observatories, the more accurate the localization. In the field of satellite-based localization, the cost required to add one satellite is huge, so the idea of increasing the number of observatories is not widely used in engineering^12^. Therefore, Vidal-Valladares M G^13^ proposed a localization algorithm based on the fusion of multiple observations, using Weighted Least Squares Batch Method (WLSB) and Weighted Least Squares Serial Method (WLSS) for the effective fusion of multiple observations. Rykała Ł and Rubiec A^14^ studied the problem based on the fusion of multiple observations and proposed a localization algorithm for time-difference-frequency-difference to cruising targets in the presence of velocity errors at the observatories. The algorithm associates the time-difference equation and frequency-difference equations observed at different moments, and combines the constraints of the cruising targets on the requested parameters to achieve localization. The rapid development in the field of satellite-based localization and the fact that satellites make multiple observations of radar emitters provide the basis for studying localization for time-difference fusion of multiple satellites^15,16^.

Gao Y^17^ pointed out that the appearance of ambiguity for time-difference is due to the ambiguity arising from pulse pairing, where the pulse repetition interval (PRI) differs by an integer multiple, and was the first to propose the concept of ambiguous localizations and real localizations. Jin S^18^ summarized the problem of accurate and fast localization of radar emitters of high pulse repetition frequency (HPRF) and proposed various methods to solve the ambiguity for time-difference. Finally, it is concluded that combining the measured time-difference with the direction information is the most effective method to achieve the accurate and fast localization of the radar emitters of HPRF. Further, Jiao G^19^ proposed an ambiguity resolution algorithm for time-difference with the assistance of direction finding information. Due to the existence of periodic subpeaks in the multi-hop correlation function leading to periodic ambiguity in TDOA estimation, Vidal R^20^ proposed the time-difference averaging single-hop (TASH) and the envelope fitting correlation function (EFCF) to eliminate the ambiguity for time-difference. After Bayesian filtering, the real trails will be filtered out from the many false trails. However, the computational complexity of the algorithm increases as the number of filtering increases. Therefore, the literature reduces the computational effort of trails splitting (TS) by pruning low-weighted trails, but this also causes incorrect localization and tracking^21,22^.

Liang J et al.^23^ discussed passive localization in distributed multiple input multiple output (MIMO) radar based on Lagrangian programming neural network (LPNN). By comparing with CRLD through computer simulation, the local stability and optimality of the localization algorithm have been demonstrated. Li Y et al.^24^ proposed a genetic algorithm (GA) based on station coding to study distance scheduling of multiple satellites. This method incorporates random selection, greedy selection, and wheel selection to address priority constraints and improve localization performance. Wang E et al.^25^ proposed a satellite-selection algorithm based on particle swarm optimization (PSO), which reduces the computational complexity when multiple satellites are running simultaneously. The Adaptive Simulated Annealing Particle Swarm Optimization (ASAPSO) method was used to improve the algorithm to prevent convergence to local minima. Kassas Z M et al.^26^ proposed a framework for low Earth orbit (LEO) satellite orbit prediction using closed-loop machine learning. The results showed that in a 4.05-kilometer journey, the traditional method achieved the error of 0.176 km, while this method achieved the error of 0.018 km. Zhang Y et al.^27^ analyzed the impact mechanism of orbit altitude, orbit inclination, number of satellites, and simulation period on accuracy, and established a new multi-layer feedforward neural network weighted joint prediction model. Li J et al.^28^ established an Attention based Long Short Term Memory Neural Network (AttLSTM) model for satellite time difference prediction. Compared with LSTM and quadratic polynomial (QP) models, AttLSTM improved prediction accuracy by 26.1% and 38.4%, 29.1% and 43.1% at 12 h and 24 h, respectively. Xu B et al.^29^ proposed a novel tracking algorithm based on an improved generalized regression neural network (GRNN) and Kalman filter (KF), and introduced cooperative co-evolutionary genetic algorithm (CCGA) to improve performance. Zhen et al.^30^ proposed a high repetition pulse deblurring method based on Residual Embedding Sequential Transformer Network (RESeTNet). Even under high repetition rate conditions of 600 kHz, RESeTNet can achieve an accuracy of 97.5% for unambiguous localization.

In summary, several key issues still need to be explored. (1) Time-difference fusion based on different observation moments. Multiple satellites at different moments will produce multiple observations of the same radar emitter, and how to effectively use the time-difference to further improve the accuracy of localization is an important issue in engineering applications. (2) Ambiguity for time-difference occurs when radar emitters of HPRF are located and tracked. Multiple pulse pairings appear in the time-difference window of radar emitters of HPRF, resulting in a large number of ambiguous localizations, leading to erroneous localization results. Therefore, how to solve ambiguity for time-difference fusion in localization and tracking is also the key issues.

**Fig. 1:**
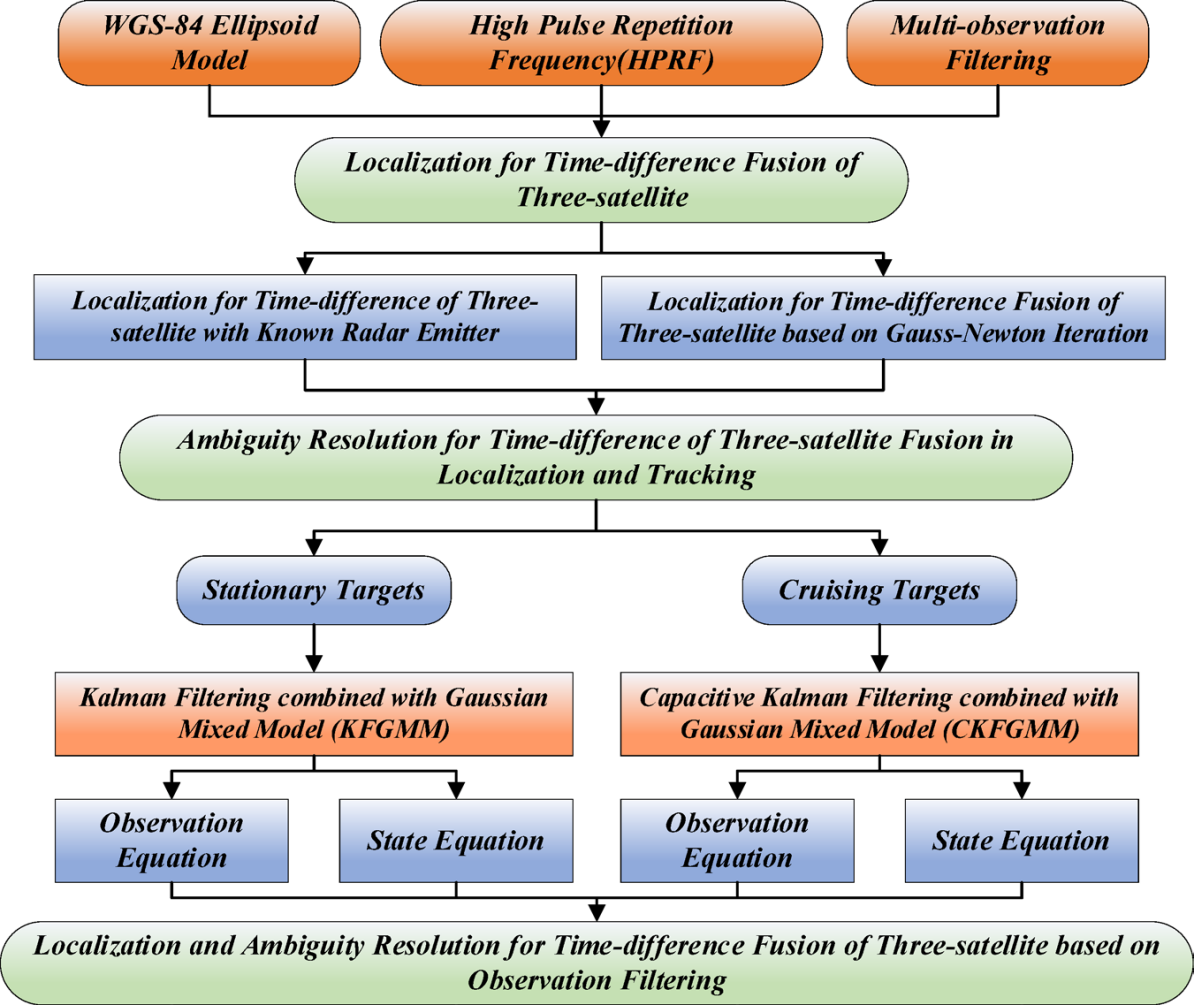
Research structure.

The structure of this paper is shown in Fig. 1. To improve the accuracy of localization for time-difference fusion, combining the time-difference equations and elevation observation equations, constructing the cost function in the overdetermined case, and the localization algorithm for time-difference fusion based on Gaussian-Newton iteration for solving nonlinear least squares problems is proposed. To resolve the ambiguity for time-difference fusion in localization and tracking, the mathematical model of the ambiguity is developed to calculate the time-difference window and ambiguous time-difference, and the Gaussian mixture model is used to approximate the measurement of ambiguity for time-difference. Meanwhile, based on the WGS-84 ellipsoidal model, the Kalman filtering combined with Gaussian mixture model (KFGMM) is proposed as the ambiguity resolution algorithm for time-difference fusion for stationary targets, and the capacitive Kalman filtering combined with Gaussian mixture model (CKFGMM) is proposed for cruising targets.

## Localization for time-difference fusion of three-satellite based on gaussian-newton iteration

### Localization for time-difference of three-satellite with known radar emitters

To study the localization for time-difference fusion of three satellites based on Gaussian-Newton iteration, the suitable initial value of the iteration needs to be chosen. Therefore, based on the WGS-84 ellipsoidal model, the results of localization for time-difference of three-satellite with known radar emitters are used as the initial value of Gaussian-Newton iteration. Further, considering the existence of multiple sets of time-difference equations for multiple observation filtering, the cost function for the overdetermined case needs to be constructed to solve and the Gaussian-Newton iteration is chosen to solve the nonlinear least squares estimation. This is because the Gaussian-Newton iteration is computationally simpler compared to the Newton-Raphson iteration, which does not require solving the second-order Jacobi matrix.

#### Mathematical model for localization

**Fig. 2:**
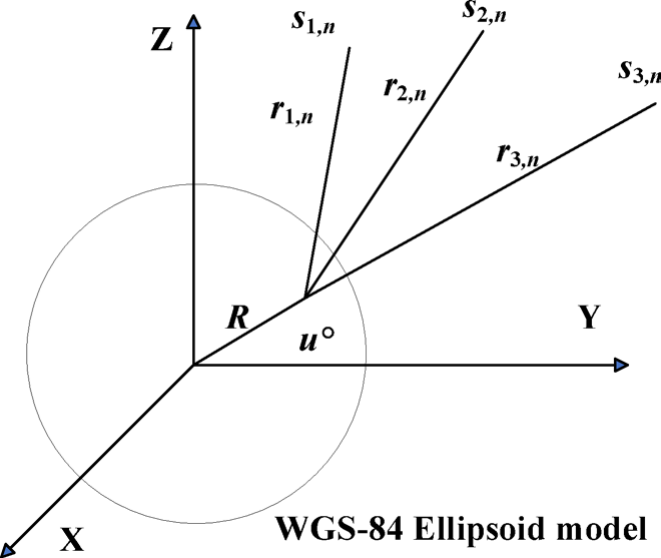
Mathematical model of localization at *n*-th moment.

The mathematical model is established as shown in Fig. 2. *R* is the sum of the Earth’s radius and the true elevation of the radar emitter, and *r* is the RODA between the radar emitter and the satellites. The stationary radar emitter on Earth is ${u^o}={[x,y,z]^T}$ and the position of satellite under *n*-th moment is ${s_{i,n}}={\left[ {{x_{i,n}},{y_{i,n}},{z_{i,n}}} \right]^T}=s_{{i,n}}^{o}+\Delta {s_{i,n}}(i=1,2,3)$, where $s_{{i,n}}^{o}={\left[ {x_{{i,n}}^{o},y_{{i,n}}^{o},z_{{i,n}}^{o}} \right]^T}$ is the real but unknown position of the satellite and $\Delta {s_{i,n}}$ is the corresponding random error of the position^31^. Assuming that ${s_n}={\left[ {s_{{1,n}}^{T},s_{{2,n}}^{T},s_{{3,n}}^{T}} \right]^T}=s_{n}^{o}+\Delta {s_n}$, where $s_{n}^{o}={\left[ {s_{{1,n}}^{{oT}},s_{{2,n}}^{{oT}},s_{{3,n}}^{{oT}}} \right]^T}$, and the Gaussian random vector with covariance matrix ${Q_{s,n}}=E\left[ {\Delta {s_n}\Delta s_{n}^{T}} \right]$ is defined as $\Delta {s_n}$.

Using the satellite *s*_1,*n*_ as the reference satellite, the TDOA between two other satellites (*s*_*i*,*n*_ (*i* = 2, 3)) observed at *n*-th moment is defined as *d*_*i*1,*n*_ :

1 $${d_{i1,n}}=\frac{{{r_{i1,n}}}}{c}=\frac{{r_{{i,n}}^{o}+{\omega _{i,n}}}}{c}=\frac{{r_{{i,n}}^{o} - r_{{1,n}}^{o}+{\omega _{i1,n}}}}{c}$$

Where *c* is the known signal propagation speed, ${r_{i1,n}}=c{d_{i1,n}}(i=2,3)$ is the RDOA between the radar emitter to *s*_*i*,*n*_ and to *s*_1,*n*_, $r_{{i1,n}}^{o}$ is the true RDOA, $\frac{{{\omega _{i1,n}}}}{c}$ is the measurement noise of the TDOA^32^, $r_{{i,n}}^{o}(i=1,2,3)$ is the true distance between the radar emitter and *s*_*i*,*n*_, *u*^*o*^ is the true position of the radar emitter, and *s*^*o*^_*i*,*n*_ is the true position of the *i*-th satellite at the *n*-th time.

2 $$r_{{i,n}}^{o}=\left\| {{u^o} - s_{{i,n}}^{o}} \right\|=\sqrt {{{\left( {x - x_{{i,n}}^{o}} \right)}^2}+{{\left( {y - y_{{i,n}}^{o}} \right)}^2}+{{\left( {z - z_{{i,n}}^{o}} \right)}^2}} (i=1,2,3)$$

Knowing the priori knowledge that the radar emitter is on the Earth’s surface, defining *R* as the sum of the Earth’s radius and the real elevation of the radar emitter, and the Earth equation under the positive spherical model can be obtained as follows:

3 $${R^2}={u^{oT}}{u^o}$$

To facilitate the calculation, the distance-difference is obtained by multiplying the measured TDOA with *c*. That is, the distance-difference observed at this moment is ${m_{t,n}}={\left[ {{r_{21,n}},{r_{31,n}}} \right]^T}$, and the error is $\Delta {m_{t,n}}={m_{t,n}} - m_{{t,n}}^{o}$, where $m_{t,n}^o = {\left[ {r_{_{21,n}}^o,r_{31,n}^o} \right]^T}$, and the Gaussian random vector with covariance matrix ${Q_{t,n}}$ is defined as $\Delta {m_{t,n}}$The elevation information of the radar emitter is added to the observation equation, which is expressed as ${m_h}=m_{h}^{o}+\Delta {m_h}$, where ${m_h}={(R+\Delta h)^2}$, and the Gaussian random variable with variance ${Q_h}$ is defined as the elevation error $\Delta h$. Neglecting the second-order error, $\Delta {m_h}=2R\Delta h$ is a Gaussian random variable with variance $4{R^2}{Q_h}$.

In summary, the expression of all the measurements is ${m_n}={\left[ {m_{{t,n}}^{T},{m_h}} \right]^T}=m_{n}^{o}+\Delta {m_n}$, $m_{n}^{o}$ is the real value of the measurements. Assuming that the time-difference observations and elevation observations are independent of each other, the Gaussian random error with covariance matrix $${Q_{m,n}}=\left[ {\begin{array}{*{20}{c}} {{Q_{t,n}}}&{{O_{2 \times 1}}} \\ {{O_{1 \times 2}}}&{4{R^2}{Q_h}} \end{array}} \right]$$ is defined as $\Delta {m_n}$. The position of satellite and velocity errors are independent of the observation errors. Firstly, this assumption can simplify mathematical models and subsequent calculations. This makes the nonlinear solution of localization easier to handle. Secondly, in practical applications, the cross-correlation between time-difference observations and elevation observations is relatively small compared to their respective variances. This assumption can minimize the impact of cross-correlation on overall accuracy. Thirdly, the assumption of independence is often used as a first-order approximation to facilitate algorithm design and engineering implementation.

#### Cramér-Rao lower bound (CRLB) of localization

The important criterion to evaluate the localization algorithm is whether the localization error can reach the Cramér-Rao Lower Bound (CRLB). In this study, it is assumed that the measurement error follows a multivariate Gaussian distribution, which is a common and reasonable assumption in many practical applications. Firstly, the mean and variance of Gaussian distribution can be directly estimated from the data, and given the mean and variance, Gaussian distribution is the maximum entropy distribution^33^. Secondly, in practical applications of signal processing and localization systems, measurement errors typically follow an approximate Gaussian distribution^33^. Thirdly, according to the central limit theorem, when the observed data is composed of the sum of a large number of independent random variables, the distribution of the observed data tends towards a Gaussian distribution^34^. Considering the localization error of the satellite, the unknown parameter $\theta ={\left[ {{u^{oT}},s_{n}^{{oT}}} \right]^T}$ and the observation vector $v={\left[ {m_{n}^{T},s_{n}^{T}} \right]^T}$ are set, and the probability density function of *v* is^33,34^:

4 $$\ln p(v;\theta )=\ln p\left( {{m_n};\theta } \right)+\ln p\left( {{s_n};\theta } \right)=K - \frac{1}{2}{\left( {{m_n} - m_{n}^{o}} \right)^T}Q_{{m,n}}^{{ - 1}}\left( {{m_n} - m_{n}^{o}} \right) - \frac{1}{2}{\left( {{s_n} - s_{n}^{o}} \right)^T}Q_{{s,n}}^{{ - 1}}\left( {{s_n} - s_{n}^{o}} \right)$$

Where *K* is a constant independent of *θ*. The CRLB of *θ* can be obtained as:

5 $$CRLB(\theta )= - E{\left[ {\frac{{\ln p(v;\theta )}}{{\partial \theta \partial {\theta ^T}}}} \right]^{ - 1}}={\left[ {\begin{array}{*{20}{c}} X&Y \\ {{Y^T}}&Z \end{array}} \right]^{ - 1}}$$

Where *X*,*Y*, and *Z* are expressed in detail as follows:

6 $$X\left\{ {\begin{array}{*{20}{l}} {X = - E\left[ {\frac{{\ln p(v;\theta )}}{{\partial {u^o}\partial {u^{oT}}}}} \right] = {{\left( {\frac{{\partial m_n^o}}{{\partial {u^o}}}} \right)}^T}Q_{m,n}^{ - 1}\left( {\frac{{\partial m_n^o}}{{\partial {u^o}}}} \right)} \\ {Y = - E\left[ {\frac{{\ln p(v;\theta )}}{{\partial {u^o}\partial s_n^{oT}}}} \right] = {{\left( {\frac{{\partial m_n^o}}{{\partial {u^o}}}} \right)}^T}Q_{m,n}^{ - 1}\left( {\frac{{\partial m_n^o}}{{\partial s_n^o}}} \right)} \\ {Z = - E\left[ {\frac{{\ln p(v;\theta )}}{{\partial s_n^o\partial s_n^{oT}}}} \right] = {{\left( {\frac{{\partial m_n^o}}{{\partial s_n^o}}} \right)}^T}Q_{m,n}^{ - 1}\left( {\frac{{\partial m_n^o}}{{\partial s_n^o}}} \right) + Q_{s,n}^{ - 1}} \end{array}} \right.and{\text{ }}\left\{ {\begin{array}{*{20}{l}} {\frac{{\partial m_n^o}}{{\partial {u^o}}} = \left[ {\begin{array}{*{20}{c}} {{{\left( {{a_2} - {a_1}} \right)}^T}} \\ {{{\left( {{a_3} - {a_1}} \right)}^T}} \\ {2{u^{oT}}} \end{array}} \right]} \\ {\frac{{\partial m_n^o}}{{\partial s_n^o}} = \left[ {\begin{array}{*{20}{l}} {a_1^T}&{ - a_2^T}&{{O_{1 \times 3}}} \\ {a_1^T}&{ - a_3^T}&{{o_{1 \times 3}}} \\ {{O_{1 \times 3}}}&{{O_{1 \times 3}}}&{{o_{1 \times 3}}} \end{array}} \right]} \end{array}} \right.$$

Where ${a_i}=\frac{{{u^o} - s_{{i,n}}^{o}}}{{r_{{i,n}}^{o}}}(i=1,2,3)$, and *O*_1×3_ is a 1 × 3-dimensional 0-matrix. The CRLB of the radar emitter *u*_*o*_ can be obtained using the inverse formula of the partition matrix as^35^:

7 $$CRLB\left( {{u^o}} \right)={X^{ - 1}}+{X^{ - 1}}Y{\left( {Z - {Y^T}{X^{ - 1}}Y} \right)^{ - 1}}{Y^T}{X^{ - 1}}$$

When there is no localization error, the first term *X*^−1^ of the above equation is the CRLB of the radar emitter *u*_*o*_, and the second term represents the increase of the CRLB when there is the localization error^36^. The trace $trace\left( {CRLB\left( {{u^o}} \right)} \right)$ of the CRLB is the MSE of localization that can be achieved by unbiased estimation.

#### Localization for time-difference

Equation (1) is rewritten as ${r_{i,n}}+{r_{1,n}}={r_{i,n}}$; after replacing the real position of satellite $s_{{i,n}}^{o}$ with the position with error ${s_{i,n}}$, referring to Eq. (2), ${r_{i,n}}(i=1,2,3)$ is calculated; and both sides are squared and combined with Eq. (2) to obtain:

8 $$r_{{i1,n}}^{2}+2{r_{i,n}}{r_{1,n}}+r_{{1,n}}^{2}={(R+\Delta h)^2}+s_{{i,n}}^{T}{s_{i,n}} - 2s_{{i,n}}^{T}{u^o}(i=2,3)$$

By using Eq. (2) to expand ${r_{i,n}}$, it can be obtained that:

9 $$r_{{1,n}}^{2}={(R+\Delta h)^2}+s_{{1,n}}^{T}{s_{1,n}} - 2s_{{1,n}}^{T}{u^o}$$

Considering the elevation measurement error, the Earth equation can be obtained by subtracting Eq. (9) from Eq. (8):

10 $$r_{{i1,n}}^{2}+2{r_{i,n}}{r_{1,n}}=s_{{i,n}}^{T}{s_{i,n}} - s_{{1,n}}^{T}{s_{1,n}} - 2{\left( {{s_{i,n}} - {s_{1,n}}} \right)^T}{u^o}(i=2,3)$$

Combining Eq. (9) and Eq. (10), ${r_{i,n}}$ can be used to represent the radar emitter:

11 $${u^o}=G_{1}^{{ - 1}}h$$

Where, $${G_1} \equiv - 2\left[ {\begin{array}{*{20}{c}} {s_{{1,n}}^{T}} \\ {s_{{2,n}}^{T} - s_{{1,n}}^{T}} \\ {s_{{3,n}}^{T} - s_{{1,n}}^{T}} \end{array}} \right],\quad h \equiv \left[ {\begin{array}{*{20}{c}} { - {{(R+\Delta h)}^2} - s_{{1,n}}^{T}{s_{1,n}}}&0&1 \\ {r_{{21,n}}^{2} - s_{{2,n}}^{T}{s_{2,n}}+s_{{1,n}}^{T}{s_{1,n}}}&{2{r_{21,n}}}&0 \\ {r_{{31,n}}^{2} - s_{{3,n}}^{T}{s_{3,n}}+s_{{1,n}}^{T}{s_{1,n}}}&{2{r_{31,n}}}&0 \end{array}} \right]\left[ {\begin{array}{*{20}{c}} 1 \\ {{r_{1,n}}} \\ {r_{{1,n}}^{2}} \end{array}} \right]$$. Substituting Eq. (11) into the Eq. (3) generates a fourth order polynomial about ${r_{1,n}}$. Solving this polynomial, the localization of the radar emitter at this moment can be found by substituting the positive real roots satisfying priori knowledge back into Eq. (11).

The above solution process are established under the positive spherical model. If the radar emitter is at high latitude, the localization error will increase due to the positive spherical model. Therefore, it is necessary to convert the localization results under the positive spherical model to those under the WGS-84 ellipsoidal model using spherical iterations as follows^37^:

(1) The position estimation of the radar emitter under the positive spherical model is derived, and the initial value of latitude *B*_0_ can be obtained after coordinate transformation;

(2) Eq. (12) is used to update the local Earth radius *r*_*e*_, and localization is carried out^38^:

12 $$r_{{e,i+1}}^{2}=\frac{{{a^2}}}{{1 - {e^2}{{\sin }^2}{B_i}}}\left( {1+{e^4}{{\sin }^2}{B_i} - 2{e^2}{{\sin }^2}{B_i}} \right)$$

Where *a* = 6,378,137 m is the semi-major axis, *e* is the first eccentricity, *e*^2^ = 0.00669437999013, and *B*_*i*_ is the latitude of the radar emitter;

(3) The first two steps are repeated until $\left| {{r_{e,i+1}} - {r_{e,i}}} \right|<\varepsilon$ (*ε* indicates a sufficiently small value) with no more than 5 iterations.

#### Experimental analysis

The ephemeris data of the satellite used for the experimental analysis are generated by referring to the second generation satellite of the “White Cloud” and based on the Satellite Tool Kit (STK)^39^. The orbital altitude of three satellites is set to 1100 km, the satellite spacing is 50 ~ 110 km, and the orbital parameters are set as shown in Table 1. The orbital ephemeris moment of three satellites starts from 4:00 on August 29, 2020, the ephemeris sampling interval is set to 1 s, and 960 sets of ephemeris data can be obtained during the satellite’s transit^40^.

**Table 1:** Satellite orbital parameters.

Satellite	s_1_	s_2_	s_3_
Semi-major axis	7478.14 km	7478.14 km	7478.14 km
Eccentricity	0°	0°	0°
Inclination	63.4°	63.4°	63.4°
Longitude of the ascending node	125°	124.5°	125.5°
Argument of periapsis	0°	0°	0°
Mean anomaly at epoch	6°	5.6°	5.2°

The covariance array ${Q_{t,n}}$ of the measurement error for time-difference is set as:

13 $${Q_{t,n}}=\left[ {\begin{array}{*{20}{c}} {{{\left( {c{\delta _t}} \right)}^2}}&{0.5{{\left( {c{\delta _t}} \right)}^2}} \\ {0.5{{\left( {c{\delta _t}} \right)}^2}}&{{{\left( {c{\delta _t}} \right)}^2}} \end{array}} \right]$$

Where ${\delta _t}$ is the standard deviation of the observation noise, *c* = 3 × 10^8^ m/s, $${Q_{m,n}}=\left[ {\begin{array}{*{20}{c}} {{Q_{t,n}}}&{{O_{2 \times 1}}} \\ {{O_{1 \times 2}}}&{4{R^2}\delta _{h}^{2}} \end{array}} \right]$$; ${\delta _h}$ is the standard deviation of the observation noise of the radar emitter. The covariance array ${Q_{s,n}}$ of the localization noise is ${Q_{s,n}}=\delta _{s}^{2}{I_{9 \times 9}}$, where ${\delta _s}$ is the standard deviation of the localization noise, and ${I_{9 \times 9}}$ is a 9 × 9-dimensional unit matrix.

The localization performance of this paper under different time-differences, measurement errors and localization errors is analyzed. The radar emitter is assumed to be located at sea level with longitude and latitude of 117.8 °E and 19.6 °N, respectively, and the Earth’s semi-major axis is approximated as the initial Earth radius.

**Fig. 3:**
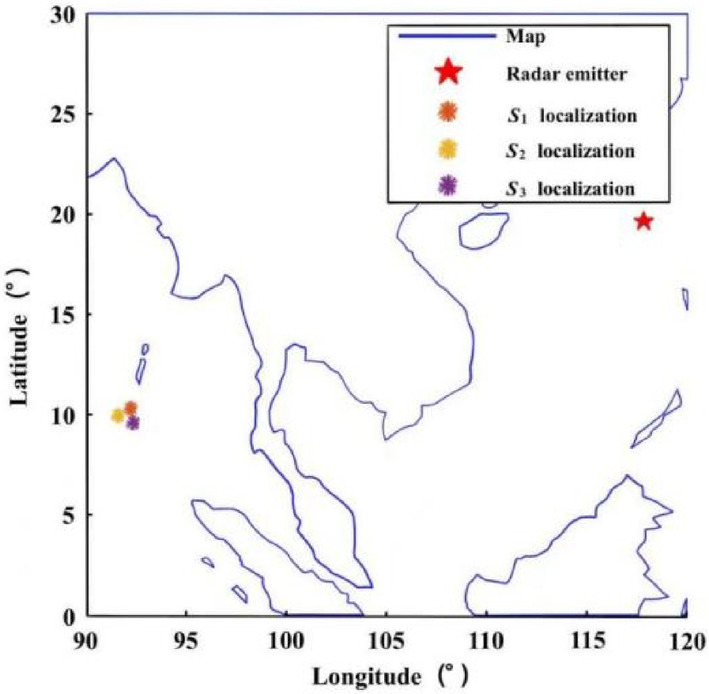
Localization scenario for time-difference.

**Fig. 4:**
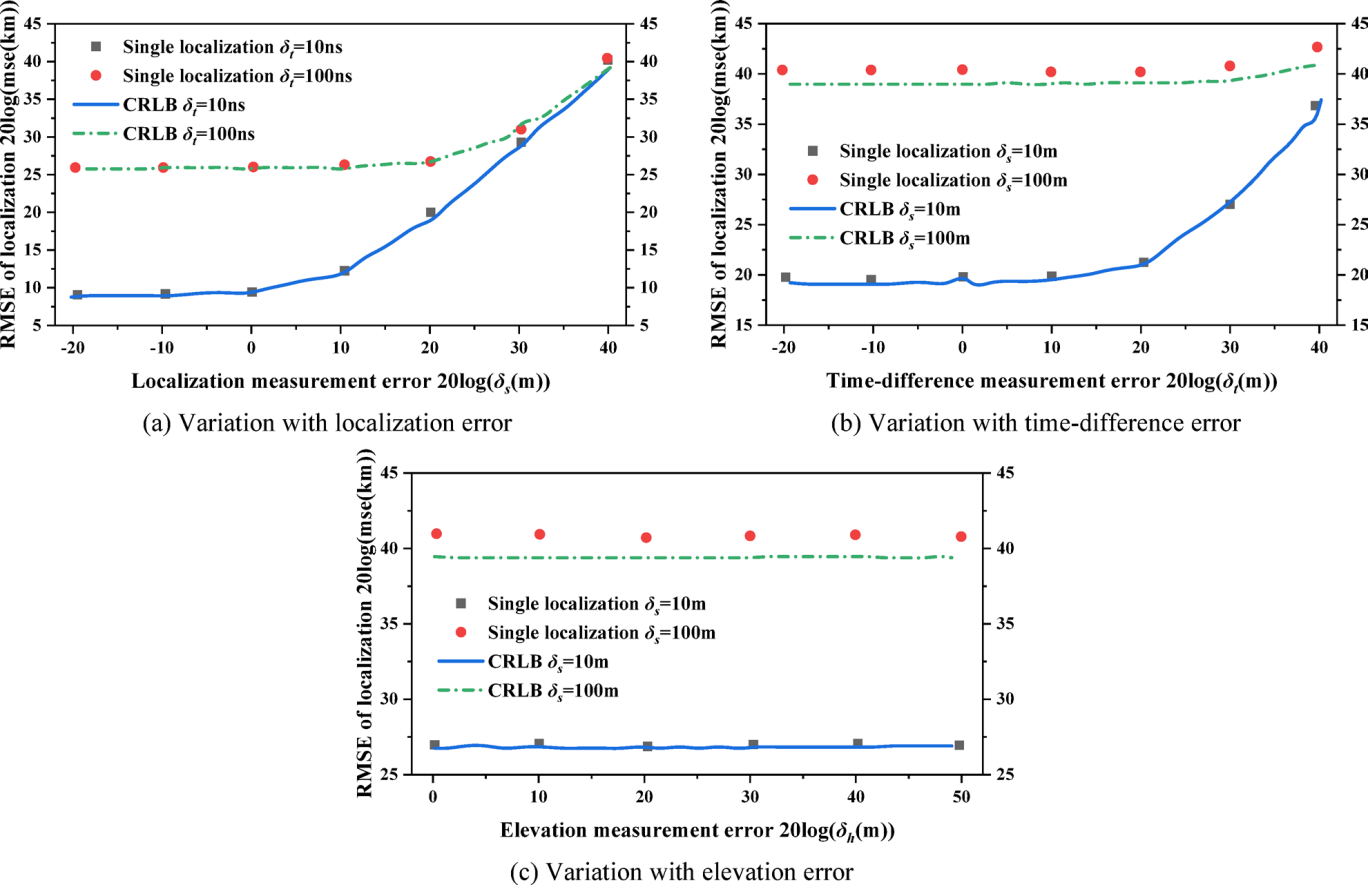
Performance of the localization algorithm for time-difference.

- The performance of the algorithm is analyzed under different localization errors. Assuming ${\delta _t}$= 10ns/100ns and ${\delta _h}$= 200 m, a set of ephemeris point data is randomly selected for localization, and the localization scenario is shown in Fig. 3. The RMSE of the localization algorithm is as follows:
- The performance of the algorithm is analyzed under different time-difference errors. Assuming ${\delta _s}$= 10 m/100m and ${\delta _h}$= 200 m, the same localization scenario shown in Fig. 3 is selected, and *M* is set to 10,000. The experimental results are shown in Fig. 4b.
- The performance of the algorithm is analyzed under different elevation measurement errors. Assuming ${\delta _s}$= 10 m/100m and ${\delta _t}$= 100ns, the same localization scenario shown in Fig. 3 is selected, and *M* is set to 10,000. The experimental results are shown in Fig. 4c.

 

14 $$RMSE=\sqrt {\frac{1}{M}\sum\limits_{{m=1}}^{M} {{{\left\| {{u^o} - {{\widehat {u}}_m}} \right\|}^2}} }$$

Where the number of Monte Carlo simulation *M* is set to 10,000 and ${\widehat {u}_m}$ is the estimated value of each localization. The experimental results are shown in Fig. 4a.

Figure 4(a) shows that the algorithm in this paper can reach the theoretical lower bound of localization error, i.e., CRLB, when the time-difference error and elevation error are constant and within a reasonable range; as the localization error gradually increases, the performance of the localization gradually deviates from the CRLB. Figure 4c is similar to Fig. 4b, and the results show that the localization performance of the algorithm reaches the CRLB when the localization error is small; and the algorithm localization performance deviates slightly from the CRLB when the position error is large. Overall, the elevation error does not have a significant impact on the localization performance, while the time-difference error and localization error has a greater impact on the localization performance, among which the gradual increase of the localization error will make the localization performance of the algorithm deviate from the CRLB. In summary, the results of the localization for time-difference of three-satellite can be used as the initial value of Gaussian-Newton iteration.

### Localization for Time-difference fusion of three satellites

#### Mathematical model of fusion localization

The above is a localization model for the radar emitter *u*^*o*^ by three satellites at *n*-th moment, and here the same *u*^*o*^ is further localized at *N* different moments respectively. Assuming that $s={\left[ {s_{{1,1}}^{T},s_{{2,1}}^{T},s_{{3,1}}^{T}, \ldots ,s_{{1,n}}^{T},s_{{2,n}}^{T},s_{{3,n}}^{T}, \ldots ,s_{{1,N}}^{T},s_{{2,N}}^{T},s_{{3,N}}^{T}} \right]^T}$ is the positions of three satellites at *N* different observation moments; $\Delta s=s - {s^o}$ is the position error, where ${s^o}={\left[ {s_{{1,1}}^{{oT}},s_{{2,1}}^{{oT}},s_{{3,1}}^{{oT}}, \ldots ,s_{{1,n}}^{{oT}},s_{{2,n}}^{{oT}},s_{{3,n}}^{{oT}}, \ldots ,s_{{1,N}}^{{oT}},s_{{2,N}}^{{oT}},s_{{3,N}}^{{oT}}} \right]^T}$. The Gaussian random vector with covariance matrix ${Q_s}$ is defined as $\Delta s$^41^.

The distance-difference under *N* observations is expressed as ${m_t}={\left[ {m_{{t,1}}^{T}, \ldots ,m_{{t,n}}^{T}, \ldots ,m_{{t,N}}^{T}} \right]^T}=m_{t}^{o}+\Delta {m_t}$ and $m_{t}^{o}$ is the real value of the distance-difference^42^. Assuming that the observations between different moments are independent of each other, the Gaussian random vector with covariance matrix ${Q_t}=diag\left\{ {{Q_{t,1}}, \ldots ,{Q_{t,n}}, \ldots ,{Q_{t,N}}} \right\}$ is $\Delta {m_t}$. Similarly, the elevation information of the radar emitter is also added into the observation equation, then the expression of its combined distance-difference and elevation information is $m={\left[ {m_{t}^{T},{m_h}} \right]^T}={m^o}+\Delta m$, and *m*^*o*^ is the real measurement^43^. Providing that the time-difference observations and elevation observations are independent of each other, $\Delta m$ is the Gaussian random vector with covariance matrix $${Q_m}=\left[ {\begin{array}{*{20}{c}} {{Q_t}}&{{O_{2N \times 1}}} \\ {{O_{1 \times 2N}}}&{4{R^2}{Q_h}} \end{array}} \right]$$.

#### CRLB of fusion localization

Unlike the CRLB of single localization, the CRLB of fusion localization needs to consider *N* observation errors and localization errors of three satellites at *N* different moments^44^. Therefore, it is assumed that the unknown parameter is $\theta ={\left[ {{u^{oT}},{s^{oT}}} \right]^T}$ and the observation vector is $v={\left[ {{m^T},{s^T}} \right]^T}$. The derivation of the CRLB of fusion localization is the same as Eqs. (4)-(7), and it should be noted that $\frac{{\partial {m^o}}}{{\partial {u^o}}}$ and $\frac{{\partial {m^o}}}{{\partial {s^o}}}$ are calculated in detail as follows^45^:

assuming $${C_n}=\frac{{\partial m_{{t,n}}^{o}}}{{\partial {u^o}}}=\left[ {\begin{array}{*{20}{l}} {{{\left( {{a_2} - {a_1}} \right)}^T}} \\ {{{\left( {{a_3} - {a_1}} \right)}^T}} \end{array}} \right]$$ and $${D_n}=\frac{{\partial m_{{t,n}}^{o}}}{{\partial s_{n}^{o}}}=\left[ {\begin{array}{*{20}{l}} {a_{1}^{T}}&{ - a_{2}^{T}}&{{O_{1 \times 3}}} \\ {a_{1}^{T}}&{ - a_{3}^{T}}&{{O_{1 \times 3}}} \end{array}} \right]$$, with the same *a*_*i*_ as before:

15 $$\left\{ \begin{gathered} \frac{{\partial {m^o}}}{{\partial {u^o}}} = {\left[ {\begin{array}{*{20}{c}} {{C_1}} \\ \ldots \\ {{C_n}} \\ \ldots \\ {{C_N}} \\ {2{u^{oT}}} \end{array}} \right]_{(2N + 1) \times 3}} \hfill \\ \frac{{\partial {m^o}}}{{\partial {s^o}}} = {\left[ {\begin{array}{*{20}{c}} {{D_1}}& \cdots &{{O_{2 \times 9}}}& \cdots &{{O_{2 \times 9}}} \\ \vdots & \ddots &{}&{}& \vdots \\ {{O_{2 \times 9}}}&{}&{{D_n}}&{}&{{O_{2 \times 9}}} \\ \vdots &{}&{}& \ddots & \vdots \\ {{O_{2 \times 9}}}& \cdots &{{O_{2 \times 9}}}& \cdots &{{D_N}} \\ {}&{}&{{O_{1 \times 9N}}}&{}&{} \end{array}} \right]_{(2N + 1 \times 9N}} \hfill \\ \end{gathered} \right.,n = 1,2,...,N$$

Where *O*_2×9_ and *O*_1×9*N*_ are 2 × 9-dimensional and 1 × 9 N-dimensional zero-matrices, respectively^46^.

#### Localization for Time-difference fusion of three satellites based on Gauss-Newton iteration

The localization algorithm that approximates the theoretical optimal solution by fusing multiple observations is proposed. The cost function is constructed by fusing the time-difference information at different moments and combining the elevation information of the radar emitter, and the nonlinear least squares problems are solved by Gaussian-Newton iteration, and the result is approximated to the theoretical optimal solution along the second-order derivative direction^47^.

Since both the positions of the radar emitter and the satellite are unknown, the computationally complex traditional method requires joint estimation of ${u^o}$ and ${s^o}$^48^. To simplify this problem, this paper proposes a weighted least squares algorithm that only needs to estimate the radar emitter, substituting the localization of satellite with error instead of the real localization into the cost function^49^:

16 $${\hbox{min} _{{u^o}}}{\left( {m - {{\widetilde {m}}^o}} \right)^T}{W^{ - 1}}\left( {m - {{\widetilde {m}}^o}} \right)$$

In the equation, ${\widetilde {m}^o}={\left[ {\widetilde {m}_{{t,1}}^{{oT}}, \ldots ,\widetilde {m}_{{t,n}}^{{oT}}, \ldots ,\widetilde {m}_{{t,N}}^{{oT}},m_{h}^{o}} \right]^T}$, $\widetilde {m}_{{t,n}}^{o}={\left[ {\tilde {r}_{{21,n}}^{o}\tilde {r}_{{31,n}}^{o}} \right]^T}$, where $\tilde {r}_{{i1,n}}^{o}$ can be expressed:

17 $$\tilde {r}_{{i1,n}}^{o}=\tilde {r}_{{i,n}}^{o} - \tilde {r}_{{1,n}}^{o}=\left\| {{u^o} - {s_{i,n}}} \right\| - \left\| {{u^o} - {s_{1,n}}} \right\|(i=2,3;n=1,2, \ldots ,N)$$

The above equation is the process of solving the nonlinear least squares estimation. If the localization of the radar emitter *u*^*o*^ at the *k*-th iteration is estimated to be ${\hat {u}_k}$, then the result of the *k* + 1-th iteration is:

18 $${\widehat {u}_{k+1}}={\widehat {u}_k}+{\left( {J_{k}^{T}{W^{ - 1}}{J_k}} \right)^{ - 1}}J_{k}^{T}{W^{ - 1}}\left( {m - {{\widetilde {m}}^o}} \right)$$

In the above equation, the weighting matrix is $W={Q_m}+D{Q_s}{D^T}$; the mathematical expression of *D* is consistent with $\frac{{\partial {m^o}}}{{\partial {s^o}}}$, and the real localization *s*^*o*^ of the satellite is replaced by the localization with error *s*; the real localization *u*^*o*^ of radar emitter is replaced by the result of the previous iteration ${\widehat {u}_k}$; *J*_*k*_ is the Jacobi matrix:

19 $${J_k}={\left. {\frac{{\partial {{\widetilde {m}}^o}}}{{\partial {u^o}}}} \right|_{{u^o}={{\widehat {u}}_k}}}$$

The previous localization estimation for time-difference are selected as the initial value of the iterations. The previous spherical iteration method is also used to convert the localization results under the positive spherical model to the WGS-84 ellipsoidal model to improve the localization accuracy.

#### Experimental analysis

The performances between the algorithm in this paper and other methods for fusion localization methods are compared. The covariance array ${Q_m}=diag\left\{ {{Q_{t,1}}, \ldots ,{Q_{t,n}}, \ldots ,{Q_{t,N}},4{R^2}{Q_h}} \right\}$ of measurement error is a 2 *N* + 1-dimensional square matrix, and the covariance array ${Q_s}=diag\left\{ {{Q_{s,1}}, \ldots ,{Q_{s,n}}, \ldots ,{Q_{s,N}}} \right\}$ of the localization error is a *9 N*-dimensional square matrix.

**Fig. 5:**
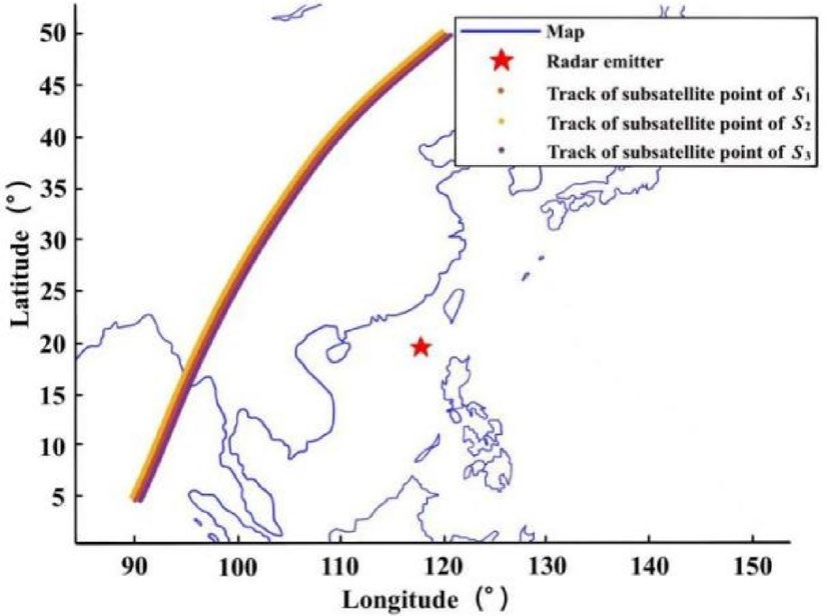
Localization scenario time-difference fusion.

**Fig. 6:**
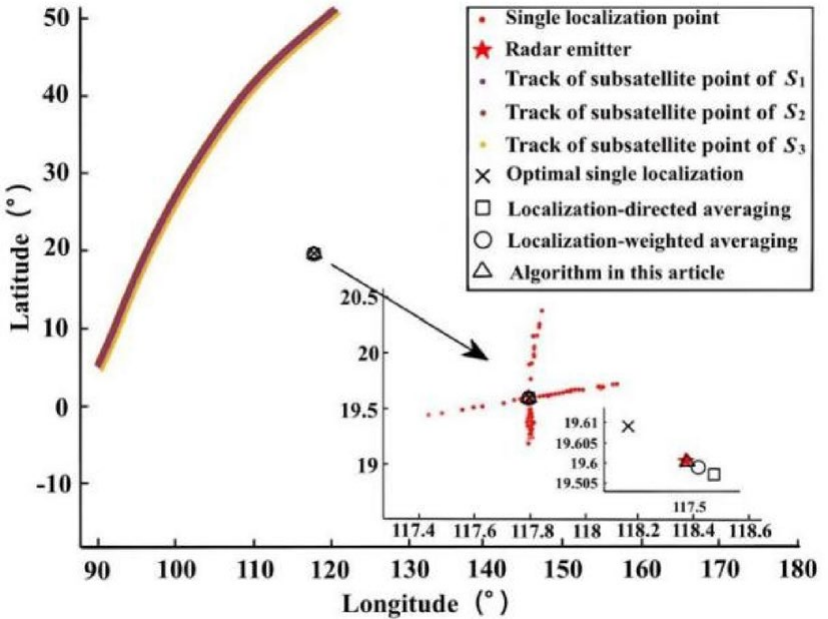
Distribution of localizations.

Assuming the position of the radar emitter is (117.8°E, 19.6°N), ${\delta _t}$= 0.2µs, ${\delta _s}$= 50 m, ${\delta _h}$= 200 m, the fusion localization scenario of 960 sets of ephemeris point data is shown in Fig. 5. The localization result when the point under the main star is closest to the radar emitter is the optimal single localization^50^; the direct average result of all single localizations is the localization-directed averaging^51^; the weighted average result of all single localizations is the localization-weighted averaging^52^. The *M* is set to 1000, and the localization results are shown in Fig. 6. From the distribution of the localizations, the single localization points are distributed in an X-shape centered on the real localization of the radar emitter. From the results of the four fusion localization methods, the localizations of the algorithm in this paper are the closest to the real localization of the radar emitter, followed by the localization-directed averaging and the localization-weighted averaging, and the optimal single localization is the worst.

**Fig. 7:**
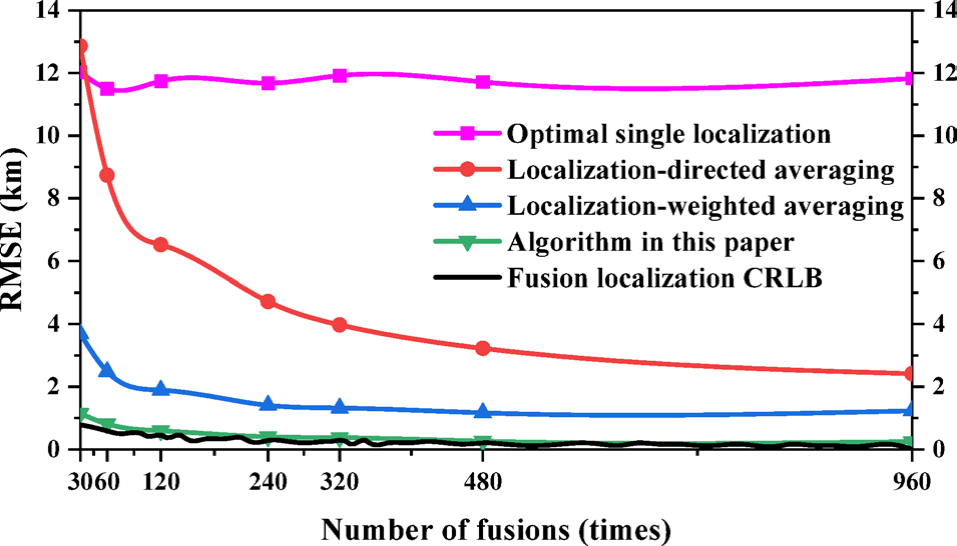
Performance comparison of different fusion localization methods (more number of fusion).

RMSE was used to compare the performances between different fusion localization methods. The fusion times *N* are taken as 30, 60, 120, 240, 320, 480 and 960 for uniform sampling, and the sampled data are used for fusion localization, are shown in Fig. 7. The localization accuracy of all three fusion localization methods except the optimal single localization gradually increases as the number of fusion gradually increases. Under the same conditions, the algorithm in this paper can achieve the CRLB of fusion localization with the best performance, followed by the localization-weighted averaging; at 30 fusions, the performance of the localization-directed averaging is inferior to the optimal single localization, and as the number of fusion increases to 60 times, the performance of the localization-directed averaging starts to be better than the optimal single localization, but always inferior to the localization-weighted averaging and the algorithm in this paper.

**Fig. 8:**
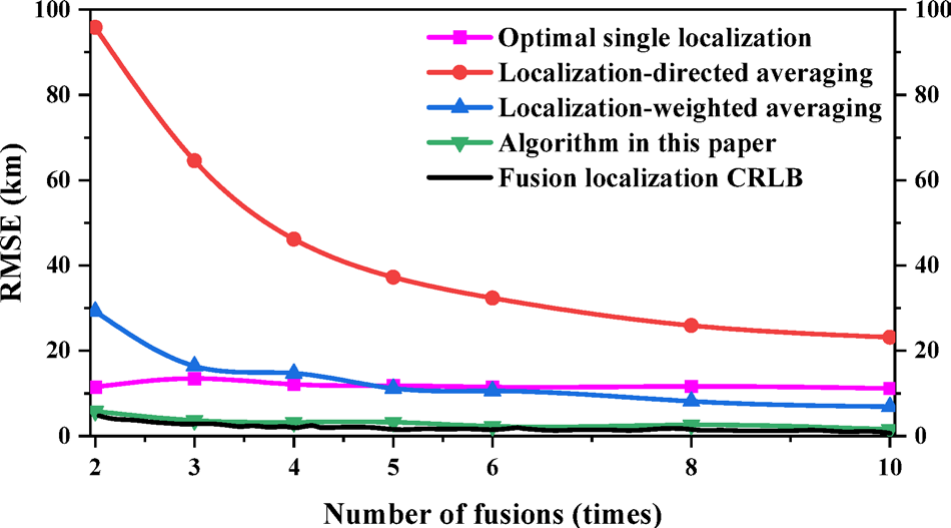
Performance comparison of different fusion localization methods (Less number of fusion).

Considering the complex and variable space electromagnetic environment in engineering applications, the signal of radar emitter may be intercepted only a few times. Therefore, the data are uniformly sampled when the fusion times *N* are 2, 3, 4, 5, 6, 8 and 10, as shown in Fig. 8. The analysis results show that the performance of the algorithm in this paper can still achieve the CRLB of fusion localization when the number of fusion is small. When the number of fusion is small, the performance of the optimal single localization is better than the localization-weighted averaging and the localization-directed averaging; after the number of fusion exceeds 5, the performance of the localization-weighted averaging starts to be better than the optimal single localization; while the performance of the localization-directed averaging is always the worst compared with the other three methods.

## Ambiguity resolution for Time-difference in localization based on observation filtering

### Ambiguity modeling for time-difference

**Fig. 9:**
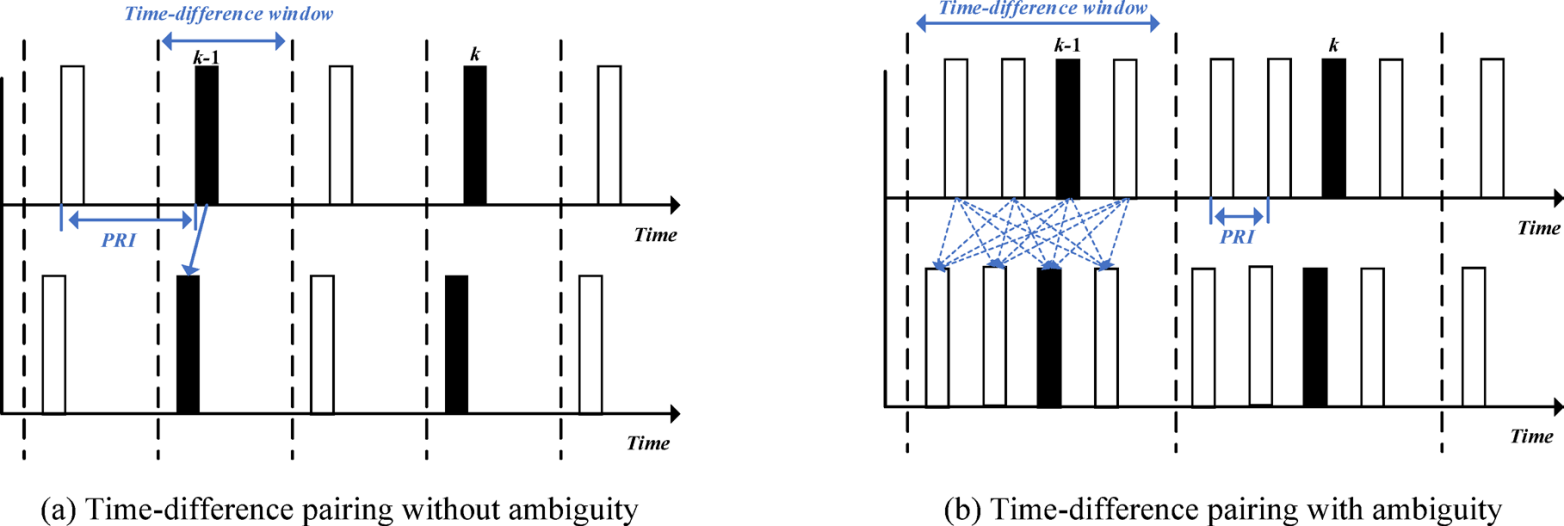
Principle of time-difference pairing.

Figure 9a shows the time-difference pairing between two satellites without ambiguity, corresponding to the top and bottom sub-plots, respectively. There is only one time difference between each pair of satellites, which can be directly used for localization. In this case, the pulse repetition interval (PRI) of the radar emitter is greater than half of the time-difference window, so there will be no pairing of multiple pulses within the time-difference window^53^. Figure 9b shows the ambiguity of time-difference pairing in the case of a high pulse repetition frequency (HPRF) radar emitter, corresponding to the top and bottom sub-plots, respectively. Due to the pulse repetition interval (PRI) being less than half of the time-difference window, multiple pulses are paired within the time-difference window, resulting in multiple possible time differences and ambiguity. This ambiguity increases the complexity of localization and requires additional algorithms to solve^53^.

**Fig. 10:**
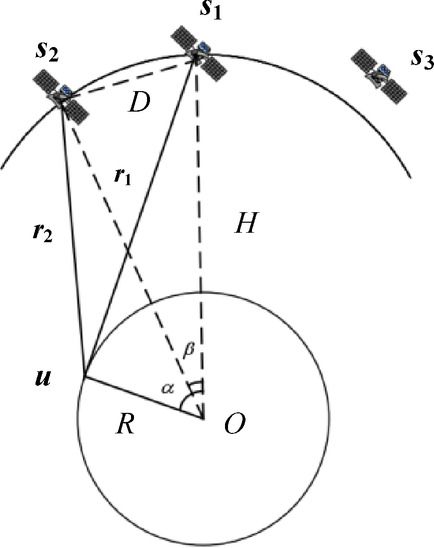
Localization model of the time-difference window.

The solution of the ambiguity for time-difference needs to calculate the time-difference window of three satellites for localization, and the three satellites need to be non-collinear with the geocentric to ensure good localization effect. Assuming that the satellite is ${s_i}(i=1,2,3)$ and the radar emitter of HPRF is *u*, the calculation model of the time-difference window of the three satellites is shown in Fig. 10.

The range of the time-difference window for *s*_1_ and *s*_2_ is $WI{N_{21}}\left( {{d_{21,\hbox{min} }},{d_{21,\hbox{max} }}} \right)=\left( {\frac{{{r_2} - {r_1}}}{c},\frac{{{r_1} - {r_2}}}{c}} \right)$, where *d*_21,*min*_ and *d*_21,*max*_ are the lower and upper bounds of the time-difference window, respectively, *u* is the tangent point between *s*_1_ and the Earth^54^. Assuming that the satellite spacing of *s*_1_ and *s*_2_ is *D*, the orbit height is *H*, and the radius of the Earth is *R*, then:

20 $${r_1}=\sqrt {{{(R+H)}^2} - {R^2}}$$

21 $$\alpha =\arccos \frac{R}{{R+H}}$$

22 $$\beta =\arccos \left( {1 - \frac{{{D^2}}}{{2{{(R+H)}^2}}}} \right)$$

23 $${r_2}=\sqrt {{{(R+H)}^2}+{R^2} - 2R(R+H)\cos (\alpha - \beta )}$$

Assuming that the PRI is known, the number of ambiguous time-difference *N*_21,*k*_ within $WI{N_{21}}\left( {{d_{21,\hbox{min} }},{d_{21,\hbox{max} }}} \right)$ can be derived from the following equation since the ambiguous time-difference differ from each other by the integer multiple of the PRI.

24 $${d_{21,\hbox{min} }} \leqslant {d_{21,{\text{ }}real{\text{ }}}}+N \cdot PRI \leqslant {d_{21,\hbox{max} }}$$

Where *d*_21,*real*_ is the real time-difference, the number of integers *N* is the number of ambiguous time-difference *N*_21,*k*_. The range of time-difference window $WI{N_{31}}\left( {{d_{31,\hbox{min} }},{d_{31,\hbox{max} }}} \right)$ and the number of ambiguous time-difference *N*_31,*k*_ of *s*_1_ and *s*_3_ are calculated as above. The numbers of ambiguous time-difference in the time-difference windows are paired with the elevation measurements to obtain a total of *N*_21,*k*_ · *N*_31,*k*_ sets of measurements, which can be substituted into the localization equation to obtain *N*_21,*k*_ · *N*_31,*k*_ localization results ${\hat u_m}(m = 1,...,{N_{21,}}_k \cdot \;{N_3}1{,_k})$^55^.

All ${\widehat {u}_m}$ must satisfy within the common visual range of the three satellites, then satisfy:

25 $${\left( {{{\widehat {u}}_m} - {s_i}} \right)^\prime }{\widehat {u}_m}<0\quad i=1,2,3$$

Finally, the results that do not satisfy the above equation are removed.

### Approximation with Gaussian mixture model

**Fig. 11:**
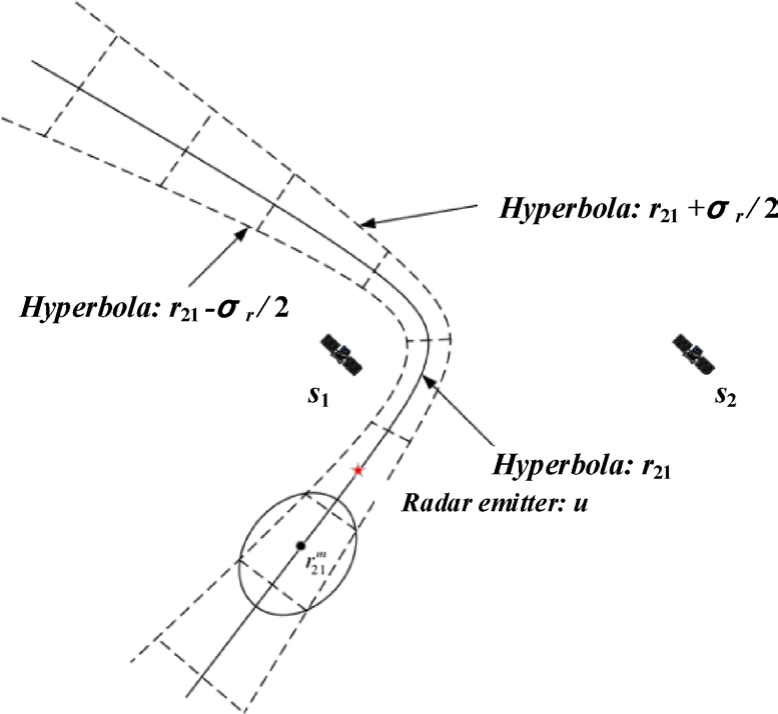
Approximation with Gaussian mixture model for distance-difference.

The time-difference measurements allow to determine the only hyperbola and are surrounded by the *σ*-area to represent the uncertainty in the location of the radar emitter. Taking *s*_1_ and *s*_2_ as examples (*s*_1_ and *s*_3_ as well), Fig. 11 gives the hyperbola for the distance-difference measurement *r*_21_ and a *σ*-region defined based on the hyperbolas *r*_21_ + *σ*_*r*_/2 and *r*_21_ - *σ*_*r*_/2^56^. All ambiguous and real measurements are segments of each Gaussian distribution on the hyperbola, with the center of the segment being the measurement mean and the covariance defined by the probability-error ellipse. Here it is assumed that each Gaussian component is independent of each other^57^.

Assuming that there are *N*_*k*_ groups of measurements at *k*-th moment, and the mean of each group of measurements is a Gaussian component of $\widehat {Z}_{k}^{n}$ and the covariance matrix of $R_{k}^{n}$. That is, the probability density function at *k*-th moment is a weighted sum of Gaussian distributions as follows:

26 $$P\left( {{Z_k}} \right)=\sum\limits_{{n=1}}^{{{N_k}}} {\mu _{k}^{n}} \frac{1}{{{{(2\pi )}^{{n_z}/2}}{{\det }^{1/2}}\left( {R_{k}^{n}} \right)}}\exp \left[ { - \frac{1}{2}{{\left( {{Z_k} - \widehat {Z}_{k}^{n}} \right)}^T}{{\left( {R_{k}^{n}} \right)}^{ - 1}}\left( {{Z_k} - \widehat {Z}_{k}^{n}} \right)} \right]$$

Where *n*_*z*_ is the dimensionality of the measurements, and the weight of each group is the same as $\mu _{k}^{n}=\frac{1}{{{N_k}}}\left( {n=1, \ldots ,{N_k}} \right)$.

### State equation and observation equation

#### For stationary targets

Based on the WGS-84 ellipsoidal model, the state vector of the radar emitter in the earth-fixed coordinate system at *k*-th moment is ${X_{E,k}}={\left[ {{x_k},{y_k},{z_k}} \right]^T}$. Since the radar emitter is a stationary target, the state equation is:

27 $${X_{E,k}}={F_{k - 1}}{X_{E,k - 1}}$$

Where, *F*_*k*−1_ is the state transfer matrix $${F_k}_{{ - 1}}=\left[ {\begin{array}{*{20}{c}} 1&0&0 \\ 0&1&0 \\ 0&0&1 \end{array}} \right]$$.

Taking all the results of ambiguous localization under *k*-th moment as measurements, assuming that the localization result is ${Z_k}={\left[ {{{\hat {x}}_k},{{\hat {y}}_k},{{\hat {z}}_k}} \right]^T}$, the observation equation is:

28 $${Z_{E,k}}={H_k}{X_k}+\Delta {m_k}$$

Where, $${H_k}=\left[ {\begin{array}{*{20}{l}} 1&0&0 \\ 0&1&0 \\ 0&0&1 \end{array}} \right]$$, the covariance array of the observation noise ${R_k}=E\left[ {\Delta {m_k}\Delta m_{k}^{T}} \right]$ can be derived from the localization error.

#### For cruise targets

Assume that the radar emitter does isometric motion on the Earth’s surface. The latitude of the radar emitter under *k*-th moment is *B*_*k*_, the longitude is *L*_*k*_, the altitude is *H*_*k*_, the velocity in the latitude direction is *V*_*N*,*k*_, and the velocity in the longitude direction is *V*_*E*,*k*_, while *V*_*H*,*k*_ is zero because of the isometric motion^58^.

Compared to the six-dimensional state vector ${X_{E,k}}={\left[ {u_{k}^{T},v_{k}^{T}} \right]^T}$ in the earth-fixed coordinate system, the motion model in this paper uses a four-dimensional state vector ${X_{G,k}}={\left[ {{B_k},{L_k},{V_{N,k}},{V_{E,k}}} \right]^T}$ in the geodetic coordinate system to improve the accuracy. Then the state equation of the isometric cruise target is:

29 $${X_{G,k}}={F_{k - 1}}{X_{G,k - 1}}+{\Gamma _{k - 1}}{w_{k - 1}}$$

Where *F*_*k*−1_ is the state transfer matrix $${F_{k - 1}}=\left[ {\begin{array}{*{20}{c}} 1&0&{\frac{T}{{\left( {{R_N}+{H_{k - 1}}} \right)}}}&0 \\ 0&1&0&{\frac{T}{{\left( {{R_E}+{H_{k - 1}}} \right)\cos {B_{k - 1}}}}} \\ 0&0&1&0 \\ 0&0&0&1 \end{array}} \right]$$; *T* is the observation interval; *R*_*N*_ is the local radius of curvature in prime vertical ${R_N}=a/\sqrt {1 - {e^2}{{\sin }^2}{B_{k - 1}}}$, where *a* = 6,378,137 m is the Earth’s semi-major axis, the square of the first eccentricity is ${e^2}=0.00669437999013$; *R*_*E*_ is the local radius of curvature in meridian ${R_E}=a\left( {1 - {e^2}} \right)/{\left( {\sqrt {1 - {e^2}{{\sin }^2}{B_{k - 1}}} } \right)^3}$. ${w_{k - 1}}={\left[ {{w_{N,k - 1}},{w_{E,k - 1}}} \right]^T}$, where ${w_{N,k - 1}}$ and ${w_{E,k - 1}}$ are the acceleration noise in the latitudinal direction and the acceleration noise in the longitudinal direction of the radar emitter at the *k-*1-th moment, respectively, and the covariance array of the acceleration noise $${Q_{k - 1}}=E\left[ {{w_{k - 1}}w_{{k - 1}}^{T}} \right]=\left[ {\begin{array}{*{20}{c}} {\sigma _{N}^{2}}&0 \\ 0&{\sigma _{E}^{2}} \end{array}} \right]$$; ${\Gamma _{k - 1}}$ is the system perturbation matrix $${\Gamma _{k - 1}}=\left[ {\begin{array}{*{20}{c}} {\frac{{{T^2}}}{{2\left( {{R_N}+{H_{k - 1}}} \right)}}}&0 \\ 0&{\frac{{{T^2}}}{{2\left( {{R_E}+{H_{k - 1}}} \right)\cos {B_{k - 1}}}}} \\ T&0 \\ 0&T \end{array}} \right]$$.

For ${X_{E,k}}={\left[ {u_{k}^{T},v_{k}^{T}} \right]^T}$, ${u_k}={\left[ {{x_k},{y_k},{z_k}} \right]^T}$ and ${v_k}={\left[ {{{\dot {x}}_k},{{\dot {y}}_k},{{\dot {z}}_k}} \right]^T}$ are the localization and velocity of the radar emitter in the earth-fixed coordinate system at *k*-th moment, respectively, then the observation equation is expressed as follows:

30 $$Z_{k}^{n}={h_E}\left( {{X_{E,k}}} \right)+\Delta {m_k}=\left\{ {\begin{array}{*{20}{l}} {{r_{21,k}}=c{t_{21,k}}={r_{2,k}} - {r_{1,k}}+\Delta {r_{21}}} \\ {{r_{31,k}}=c{t_{31,k}}={r_{3,k}} - {r_{1,k}}+\Delta {r_{31}}} \\ {{m_H}=\sqrt {u_{k}^{T}p{u_k}} +\Delta H} \end{array}} \right.$$

In the above equation, ${r_{i1,k}}(i=2,3)$ and ${t_{i1,k}}(i=2,3)$ are the distance-difference and time-difference between *s*_1_ and *s*_2_ and *s*_3_, respectively, at *k*-th moment; ${r_{i,k}}(i=1,2,3)$ is the distance between *s*_1_, *s*_2_, *s*_3_ and radar emitter; *c* is the signal propagation speed; *m*_*H*_ is the elevation measurement; $p=diag\left\{ {1,1,\frac{{{{\left( {{R_N}+{H_k}} \right)}^2}}}{{{{\left( {\left( {1 - {e^2}} \right){R_N}+{H_k}} \right)}^2}}}} \right\}$; $\Delta {r_{21}}$ and $\Delta {r_{31}}$ are the distance-difference error with variance ${\left( {c \cdot {\sigma _t}} \right)^2}$; Δ*H* is the elevation error with variance $\sigma _{H}^{2}$; and the covariance array of observation noise is $$R_{k}^{n}=E\left[ {\Delta {m_k}\Delta m_{k}^{T}} \right]=\left[ {\begin{array}{*{20}{c}} {{{\left( {c{\sigma _t}} \right)}^2}}&{0.5{{\left( {c{\sigma _t}} \right)}^2}}&0 \\ {0.5{{\left( {c{\sigma _t}} \right)}^2}}&{{{\left( {c{\sigma _t}} \right)}^2}}&0 \\ 0&0&{\sigma _{H}^{2}} \end{array}} \right]$$. The above equation is the expression of the real time-difference, and the ambiguous time-difference can be added to the real time-difference with the integer multiple of PRI. Converting the observation equation in the earth-fixed coordinate system into the observation equation in the geodetic coordinate system:

31 $${Z_k}={h_G}\left( {{X_{G,k}}} \right)+\Delta {m_k}$$

### Ambiguity resolution with Kalman filtering

**Fig. 12:**
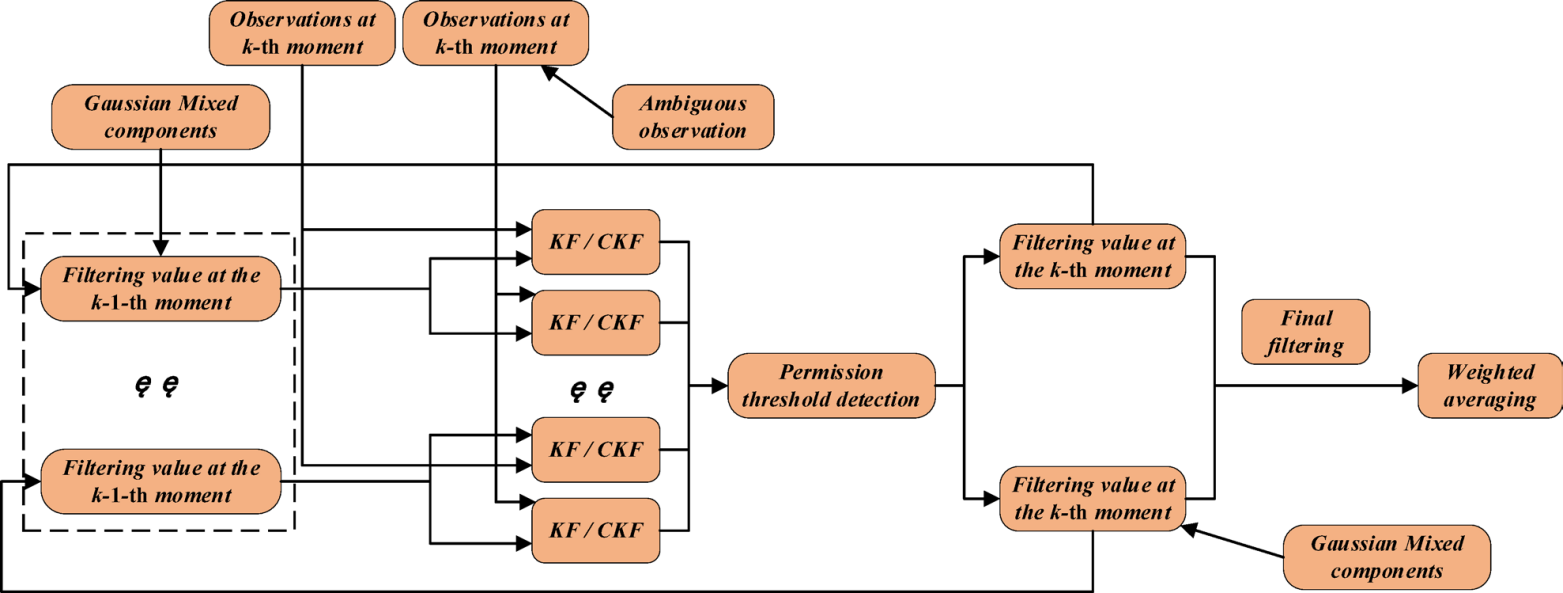
Process of ambiguity resolution.

For stationary radar emitter of HPRF, linear observation equations are constructed using ambiguous localizations and finally solved using the Kalman filtering combined with the Gaussian mixture model (KFGMM); for cruising radar emitter of HPRF, nonlinear observation equations are directly constructed using ambiguous measurements and finally solved using the nonlinear Kalman filtering combined with the Gaussian mixture model^59^. Considering that the performance of the capacitive Kalman filtering (CKF) is superior to that of the extended Kalman filtering (EKF) [60 and the unscented Kalman filtering (UKF)^61^ in solving the high-dimensional and divergence problems, and the huge computational effort of particle filtering (PF)^62^ is not required, this paper adopts the capacitive Kalman filtering combined with the Gaussian mixture model (CKFGMM) to solve the ambiguity for time-difference for the cruising radar emitter of HPRF, as shown in Fig. 12.

This process includes the following key steps: (1) Initialization. At the initial moment, all possible fuzzy localization results and their corresponding CRLB are obtained as the initial filtering values. (2) Prediction. Each filtering value is predicted using the state transition matrix *F*_*k*−1_ and the covariance matrix *Q*_*k*−1_ of process noise, and the predicted value at the *k*-th moment is obtained. (3) Update. Combining the measured values at the *k*-th moment, the Kalman gain *K*_*k*_ is calculated, and each predicted value is updated to obtain the filtered value at the *k*-th moment. (4) Pruning. The weight threshold *λ* is set, and filter values with weights less than *λ* are removed to reduce computational complexity. (5) All filter values are weighted and averaged to obtain the final localization result.

#### Stationary targets

The detailed process of fusion localization of stationary radar emitter of HPRF using KFGMM is as follows.

**Step 1**

The initial filtering value is determined. All the ambiguous localization within the visual range of the three satellites and their corresponding CRLB are obtained at the initial moment as the initial filtering values $\widehat {X}_{{E,0}}^{m}$ and $\widehat {P}_{{E,0}}^{m}$, where $m=1,...,{M_0}$. The weight of the initial component is $w_{0}^{m}=\frac{1}{{{M_0}}}$.

**Step 2**

Assuming that there are a total of ${M_{k - 1}}$ sets of filtering $\widehat {X}_{{E,k - 1}}^{m}$ and $\widehat {P}_{{E,k - 1}}^{m}$ at *k*−1-th moment, where $m=1,...,{M_{k - 1}}$, the predicted value at *k*-th moment is.

**Step 3**

The filtering gain *K*_*k*_ is calculated by combining the *N*_*k*_ sets of measurements $Z_{k}^{n},n=1,...,{N_k}$ obtained at *k*-th moment, and correct the predicted values of the target state, then the filtering state $\widehat {X}_{{E,k}}^{r}$, covariance matrix $\widehat {P}_{{E,k}}^{r}$ and corresponding weights $w_{k}^{r}$ at *k*-th moment are obtained.

**Step 4**

With *λ* as the threshold, the components with $w_{k}^{r}<\lambda$ are removed and the remaining components $\left\{ {\widehat {X}_{{E,k}}^{\prime },\hat {P}_{k}^{{{r^\prime }}},w_{k}^{\prime }} \right\}$ are substituted into the next filtering. The last filtering takes the weighted average of all filtering results after pruning as the final result.

In the above equations,$$\eta _{k}^{r} = \frac{1}{{(2\pi )^{{m/2}} \sqrt {\det \left( {\widehat{P}_{{E,kk - 1}}^{m} + R_{k}^{n} } \right)} }}$$$$\cdot \exp$$$$\left( { - \frac{1}{2}\left( {Z_{k}^{n} - \widehat{X}_{{E,k/k - 1}}^{m} } \right)^{T}}\right.$$ $$\left. {\left( {\widehat{P}_{{E,kk - 1}}^{m} + R_{k}^{n} } \right)^{{ - 1}} \left( {Z_{k}^{n} - \widehat{X}_{{E,k/k - 1}}^{m} } \right)} \right)$$, nz is the dimension of the measurements,

$$r=(m - 1){N_k}+n$$ 

32 $$\widehat {X}_{{E,k/k - 1}}^{m}=\widehat {X}_{{E,k - 1}}^{m} {\text{ }}\widehat {P}_{{E,k k - 1}}^{m}=\widehat {P}_{{E,k - 1}}^{m}$$

33 $${K_k}=\widehat {P}_{{E,k k - 1}}^{m}{\left( {\widehat {P}_{{E,k k - 1}}^{m}+R_{k}^{n}} \right)^{ - 1}}$$

34 $$\widehat {X}_{{E,k}}^{r}=\widehat {X}_{{E,k k - 1}}^{m}+{K_k}\left( {Z_{k}^{n} - \widehat {X}_{{E,kk - 1}}^{m}} \right)$$

35 $$\widehat {P}_{{E,k}}^{r}=\widehat {P}_{{E,k k - 1}}^{m} - {K_k}\widehat {P}_{{E,k k - 1}}^{m}$$

36 $$w_{k}^{r}=\frac{{w_{{k - 1}}^{m}\eta _{k}^{r}}}{{\sum\limits_{{m=1}}^{{{M_{k - 1}}}} {\sum\limits_{{r=1}}^{{{N_k}}} {w_{{k - 1}}^{m}} } \eta _{k}^{r}}}$$

37 $${\widehat {X}_{E,k}}=\sum {w_{k}^{\prime }} \cdot \widehat {X}_{{E,k}}^{{{r^\prime }}}$$

#### Cruising targets

The detailed process of localization and tracking of the cruising radar emitter of HPRF using the CKFGMM is as follows.

##### Step 1

The initial filtering value is determined. All ambiguous measurements for time-difference are obtained at the initial moment and combined with the elevation measurements to obtain all ambiguous localization and their corresponding CRLB as the initial filtering values $\widehat {X}_{{G,0}}^{m}$ and $\widehat {P}_{{G,0}}^{m}$, where $m=1,...,{M_0}$. The velocity component of the initial filtering values is set to zero, and the covariance component of initial velocity is set to $$\left[ {\begin{array}{*{20}{c}} {V_{{\hbox{max} ,N}}^{2}}&0 \\ 0&{V_{{\hbox{max} ,E}}^{2}} \end{array}} \right]$$, where $V_{{\hbox{max} ,N}}^{2}$ and $V_{{\hbox{max} ,E}}^{2}$ are the theoretical maxima of the velocity in the latitude and longitude directions, and the weight of the initial component is $w_{0}^{m}=\frac{1}{{{M_0}}}$.

##### Step 2

Assuming that there are a total of ${M_{k - 1}}$ sets of filtering $\widehat {X}_{{E,k - 1}}^{m}$ and $\widehat {P}_{{E,k - 1}}^{m}$ at *k*−1-th moment, where $m=1,...,{M_{k - 1}}$, the predicted value at *k*-th moment is.

38 $$\begin{gathered} \widehat {X}_{{G,k k - 1}}^{m}=F_{k}^{m}\widehat {X}_{{G,k - 1}}^{m} \\ \widehat {P}_{{G,k k - 1}}^{m}=F_{{k - 1}}^{m}\widehat {P}_{{G,k - 1}}^{m}F_{{k - 1}}^{{m,T}}+\Gamma _{{k - 1}}^{m}{Q_{k - 1}}\Gamma _{{k - 1}}^{{m,T}} \\ \end{gathered}$$

39 $$\chi _{{G,k}}^{{ - (i)}}=\widehat {X}_{{G,k k - 1}}^{m}+\sqrt {\widehat {P}_{{G,k k - 1}}^{m}} {\xi ^i},i=1, \ldots ,2n$$

40 $${\xi ^i}=\sqrt n {\left[ {{I_n}, - {I_n}} \right]_i}$$

41 $$\chi _{{E,k}}^{{ - (i)}}=T\left( {\chi _{{G,k}}^{{ - (i)}}} \right),i=1, \ldots ,2n$$

42 $$\widehat {\gamma }_{k}^{{(i)}}={H_E}\left( {\chi _{{E,k}}^{{ - (i)}}} \right)$$

##### Step 3

Capacitive points are constructed using the predicted values and substituted into the observation equation, where *n* represents the number of state dimensions.

43 $$\begin{gathered} \mu _{k}^{m}=\frac{1}{{2n}}\sum\limits_{{i=0}}^{{2n}} {\widehat {\gamma }_{k}^{{(i)}}} \\ S_{k}^{m}=\frac{1}{{2n}}\sum\limits_{{i=0}}^{{2n}} {\left( {\widehat {\gamma }_{k}^{{(i)}} - \mu _{k}^{m}} \right)} {\left( {\widehat {\gamma }_{k}^{{(i)}} - \mu _{k}^{m}} \right)^T}+{R_k} \\ C_{k}^{m}=\frac{1}{{2n}}\sum\limits_{{i=0}}^{{2n}} {\left( {\chi _{{G,k}}^{{ - (i)}} - \widehat {X}_{{G,kk - 1}}^{m}} \right)} {\left( {\widehat {\gamma }_{k}^{{(i)}} - \mu _{k}^{m}} \right)^T} \\ \end{gathered}$$

44 $${K_k}=C_{k}^{m}{\left( {S_{k}^{m}} \right)^{ - 1}}$$

45 $$\widehat {X}_{{G,k}}^{r}=\widehat {X}_{{G,k k - 1}}^{m}+{K_k}\left( {Z_{k}^{n} - \mu _{k}^{m}} \right)$$

46 $$\widehat {P}_{{G,k}}^{r}=\widehat {P}_{{G,k k - 1}}^{m} - {K_k}S_{k}^{m}K_{k}^{T}$$

47 $$w_{k}^{r}=\frac{{w_{{k - 1}}^{m}\eta _{k}^{r}}}{{\sum\limits_{{m=1}}^{{{M_{k - 1}}}} {\sum\limits_{{r=1}}^{{{N_k}}} {w_{{k - 1}}^{m}} } \eta _{k}^{r}}}$$

##### Step 4

Combining the *N*_*k*_ sets of measurements $Z_{k}^{n},n=1,...,{N_k}$ obtained at *k*-th moment, the filtering gain *K*_*k*_, filtering state $\widehat {X}_{{G,k}}^{r}$, covariance matrix $w_{k}^{r}$ and weights are calculated. The state is updated as follows

The $\eta _{k}^{r}$ is changed to $\eta _{k}^{r}=\frac{1}{{{{(2\pi )}^{n/2}}\sqrt {\det \left( {S_{k}^{m}} \right)} }} \cdot \exp \left( { - \frac{1}{2}{{\left( {Z_{k}^{n} - \mu _{k}^{m}} \right)}^T}{{\left( {S_{k}^{m}} \right)}^{ - 1}}\left( {Z_{k}^{n} - \mu _{k}^{m}} \right)} \right)$.

##### Step 5

The process of setting the weight threshold for pruning is the same as Step 4 for stationary targets.

The predicted mean $\mu _{k}^{m}$, the predicted covariance array $S_{k}^{m}$, and the mutual covariance array $C_{k}^{m}$ between states and observations are then calculated for the capacitive points:

As mentioned above, before the filtering starts, the real localization and all ambiguous localizations of the radar emitter have the same weight value of $w_{0}^{m}=\frac{1}{{{M_0}}}$. As the number of filtering increases, the weight of the real localization will gradually increase, even if only one filtering, its weight will be greater than the initial weight $\frac{1}{{{M_0}}}$. Therefore, setting the threshold range to $0<\lambda \leqslant \frac{1}{{{M_0}}}$ preserves the information of the real localization and significantly reduces the computational effort.

### Bayesian Cramér-Rao lower bound (BCRLB)

For dynamic estimation systems like Kalman filtering, the Bayesian Cramér-Rao Lower Bound (BCRLB) is used to measure the estimation performance of filtering. If the BCRLB at *k*−1-th moment is *BCRLB*_*k*−1_, then the Fisher information matrix *J*_*k*−1_ at *k*−1-th moment is defined as^63^:

48 $${J_{k - 1}} \equiv BCRLB_{{k - 1}}^{{ - 1}}$$

Then the Fisher information matrix *J*_*k*_ at *k*-th moment is:

49 $${J_k}=D_{{k - 1}}^{{22}} - D_{{k - 1}}^{{21}}{\left( {{J_{k - 1}}+D_{{k - 1}}^{{11}}} \right)^{ - 1}}D_{{k - 1}}^{{12}}$$

In the above Eqs^64^.,

50 $$\begin{gathered} D_{{k - 1}}^{{11}}=F_{{k - 1}}^{T}Q_{{k - 1}}^{{ - 1}}{F_{k - 1}} D_{{k - 1}}^{{12}}= - F_{{k - 1}}^{T}Q_{{k - 1}}^{{ - 1}} \\ D_{{k - 1}}^{{21}}={\left( {D_{{k - 1}}^{{12}}} \right)^T} D_{{k - 1}}^{{22}}=Q_{{k - 1}}^{{ - 1}}+H_{k}^{T}R_{k}^{{ - 1}}{H_k} \\ \end{gathered}$$

Substituting Eq. (50) into Eq. (49) yields *J*_*k*_ as:

51 $${J_k}=Q_{{k - 1}}^{{ - 1}} - Q_{{k - 1}}^{{ - 1}}{F_{k - 1}}{\left( {{J_{k - 1}}+F_{{k - 1}}^{T}Q_{{k - 1}}^{{ - 1}}{F_{k - 1}}} \right)^{ - 1}}F_{{k - 1}}^{T}Q_{{k - 1}}^{{ - 1}}+H_{k}^{T}R_{k}^{{ - 1}}{H_k}$$

To avoid the difficulty in solving the above equation due to the singular matrix of *Q*_*k*−1_, the inverse derivation is used to change the above equation to:

52 $${J_k}={\left( {{Q_{k - 1}}+{F_{k - 1}}J_{{k - 1}}^{{ - 1}}F_{{k - 1}}^{T}} \right)^{ - 1}}+H_{k}^{T}R_{k}^{{ - 1}}{H_k}$$

Where *Q*_*k*−1_ is the covariance array of the system perturbation noise at *k*−1-th moment, *F*_*k*−1_ is the state transfer matrix at *k*−1-th moment, *R*_*k*_ is the covariance array of the observation noise at *k*-th moment, ${H_k}=\frac{{\partial {h_G}\left( {{X_{G,k}}} \right)}}{{\partial X_{{G,k}}^{T}}}$. The BCRLB at *k*-th moment is obtained by inverting *J*_*k*_, and *BCRLB*_0_ is determined by initialization^65^.

## Experimental analysis

### For stationary targets

Before performing the ambiguity resolution, the distribution of the ambiguous localizations at the initial moment is analyzed. The longitude and latitude of the radar emitter are assumed to be 110.0°E and 15.0°N, respectively, and the elevation is zero. The orbital altitude of the three satellites is set to 1100 km, the orbital inclination is 63.4°, the satellite spacing is 50 ~ 110 km, and the sampling interval is *T* = 1s. The ephemeris point data at the initial moment are shown in Table 2.

**Table 2:** Ephemeris data of three satellites.

Satellite	X (km)	Y (km)	Z (km)
*s*_1_	−18.2	7446.2	689.9
*s*_2_	70.3	7450.1	643.6
*s*_3_	−36.2	7454.2	597.0

**Fig. 13:**
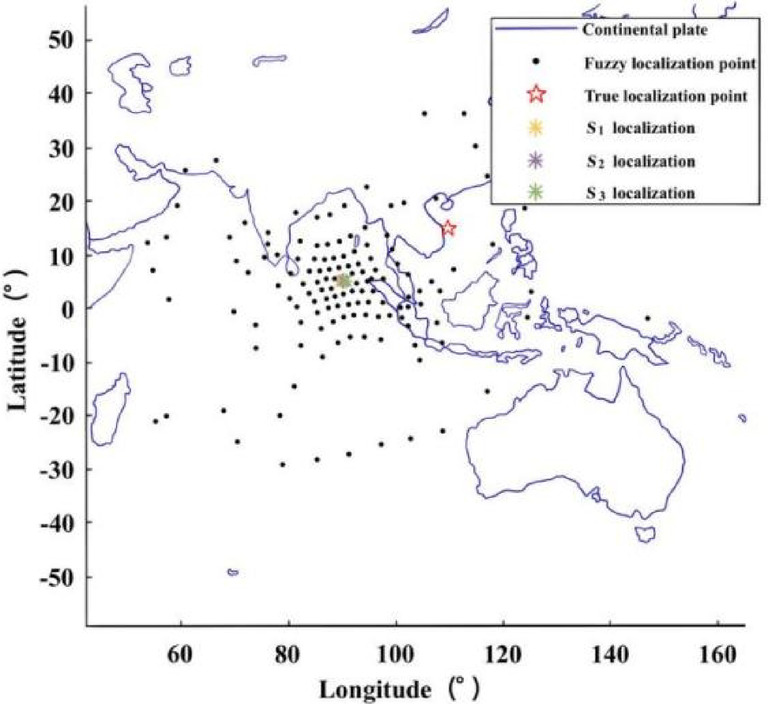
Distribution of the initial ambiguous localizations.

Assume that the measurement error for time-difference ${\delta _t}$ = 100ns, the localization error of satellite ${\delta _s}$ = 25 m, and the elevation error ${\delta _h}$ = 500 m. The time-difference window obtained in this scenario is 0.5686ms, so for the any radar emitter with PRF > 3.5k Hz will produce the ambiguity for time-difference. When the radar emitter of HPRF with PRF = 20 kHz is used, the distribution of ambiguous localizations of the three satellites at the initial moment is shown in Fig. 13. It can be seen from Fig. 13 that the number of ambiguous localizations of the three satellites is large and almost covers the whole visual range. Only one localization is close to the real localization of the radar emitter, and this localization is the result of using the real time-difference. In the latter content, all the ambiguous points of the initial localization are substituted into the Gaussian mixture model as the initial filtering value.

**Fig. 14:**
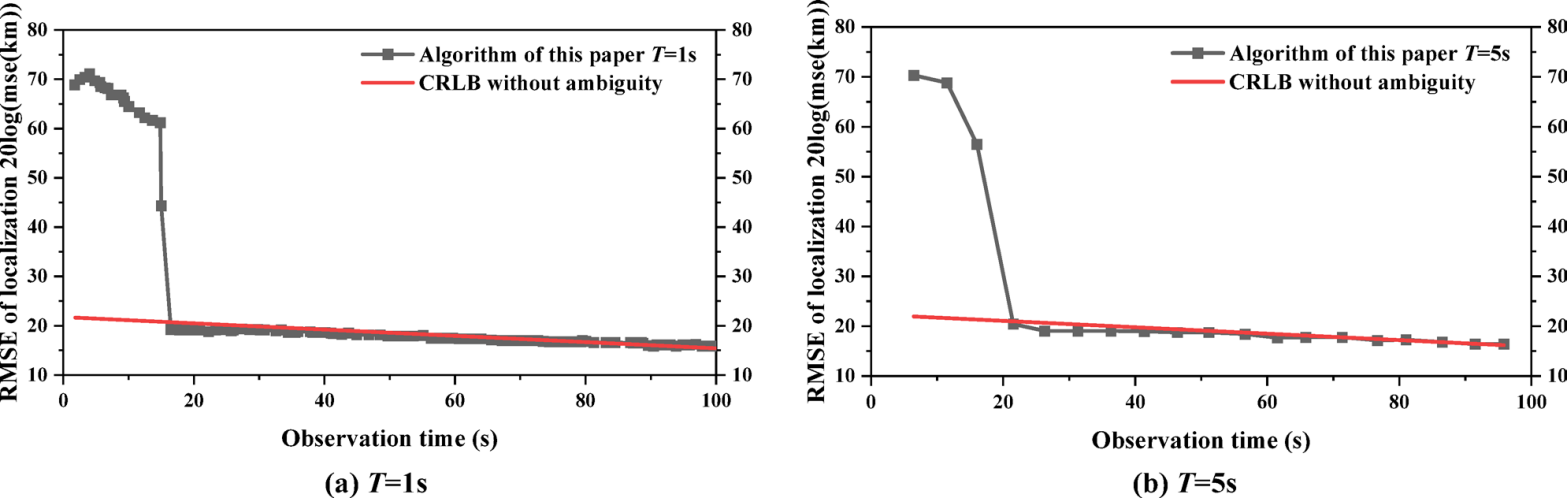
Performance of ambiguity resolution algorithm (for stationary targets).

The ambiguity resolution algorithm for stationary targets is verified. Assuming that the observation interval and the number of observation are *T* and *N*, respectively. *T* = 1 s and *N* = 100, *T* = 5 s and *N* = 20 are set for filtering, respectively, and the Gaussian weight threshold is set to *λ* = 10^−10^. Comparing the RMSE of the algorithm in this paper and the CRLB for localization without ambiguity within 100 s, *M* = 20, is shown in Fig. 14. Regardless of the observation interval, with the increase of the filtering time, the performance of the algorithm in this paper eventually reaches the CRLB for localization without ambiguity. The algorithm converges to CRLB for localization faster at *T* = 1 s than at *T* = 5s. In summary, it can be obtained that the KFGMM algorithm proposed in this paper can solve the ambiguity for time-difference effectively for stationary radar emitter of HPRF.

### For cruising targets

The low-speed targets do isometric cruising motion at 20 m/s, and the angle is *θ* = 45° east of north. Assuming that ${V_{\hbox{max} ,N}}={V_{\hbox{max} }} \cdot \sin (\theta ),{\text{ }}{V_{\hbox{max} ,E}}={V_{\hbox{max} }} \cdot \cos (\theta )$, the theoretical maximum velocity of the radar emitter is *V*_max_ = 20 m/s; *σ*_*N*_ = *σ*_*E*_ = 0.001 m/s, the number of initial ambiguous localizations *M*_0_ = 132 is obtained, so $\lambda =1/{M_0}=1/132$; *T* = 15 s, *N* = 50. The algorithm in the literature^25,28^, and^66^ uses the algorithm that compares the direction finding assistance for the ambiguity resolution for time-difference, and was used for comparison with the algorithm in this paper. The angle error of 0.1° and 0.5° are set to compare the RMSE of localization and the RMSE of velocity between the algorithm of this paper and the algorithm of literature^66^, respectively:

53 $$\left\{ {\begin{array}{*{20}{l}} {RMS{E_u}=\sqrt {\frac{1}{M}\sum\limits_{{m=1}}^{M} {{{\left\| {u - {{\hat {u}}_m}} \right\|}^2}} } } \\ {RMS{E_v}=\sqrt {\frac{1}{M}\sum\limits_{{m=1}}^{M} {{{\left\| {v - {{\widehat {v}}_m}} \right\|}^2}} } } \end{array}} \right.$$

**Fig. 15:**
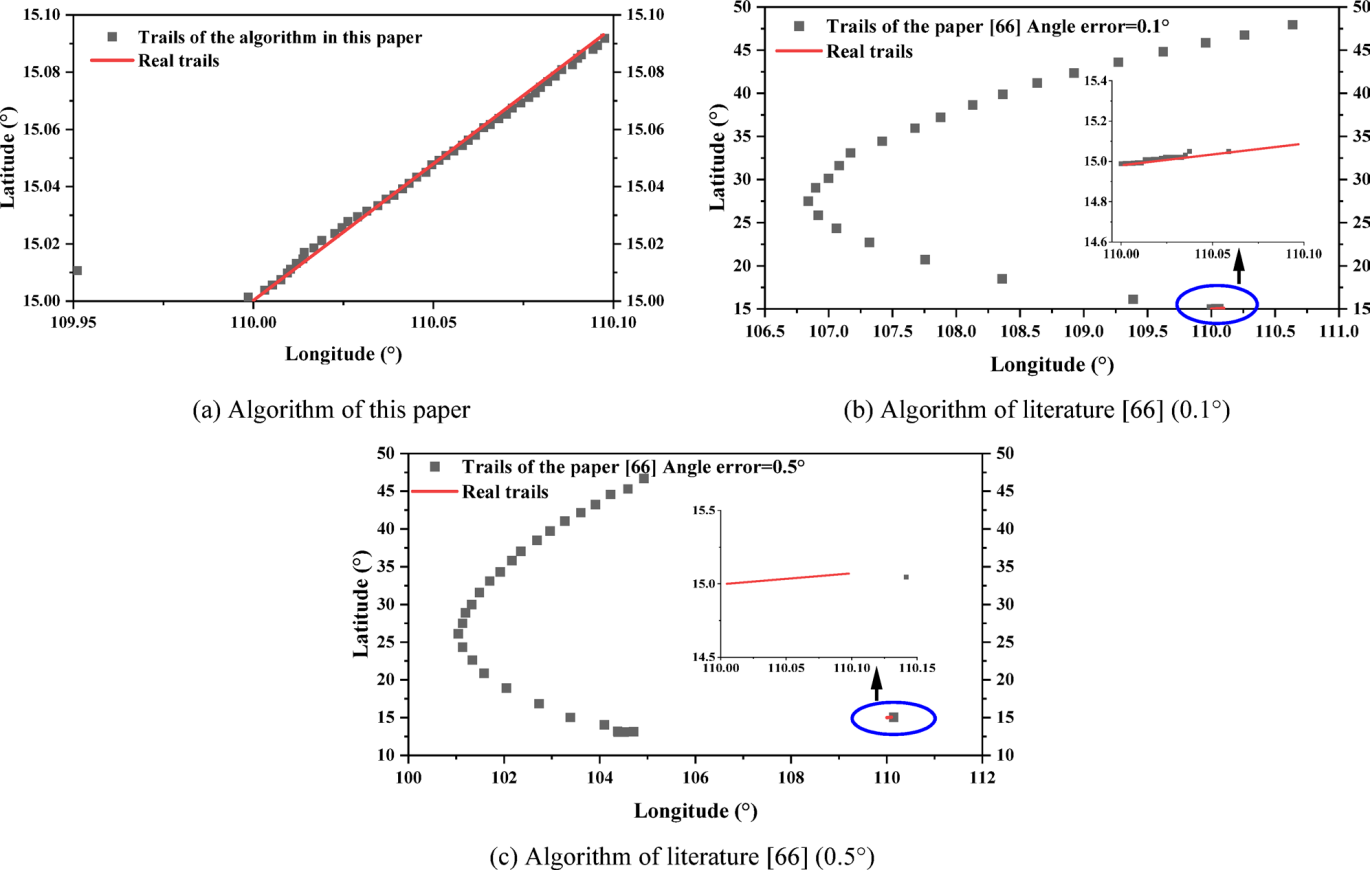
Tracking scenario for low-speed targets.

**Fig. 16:**
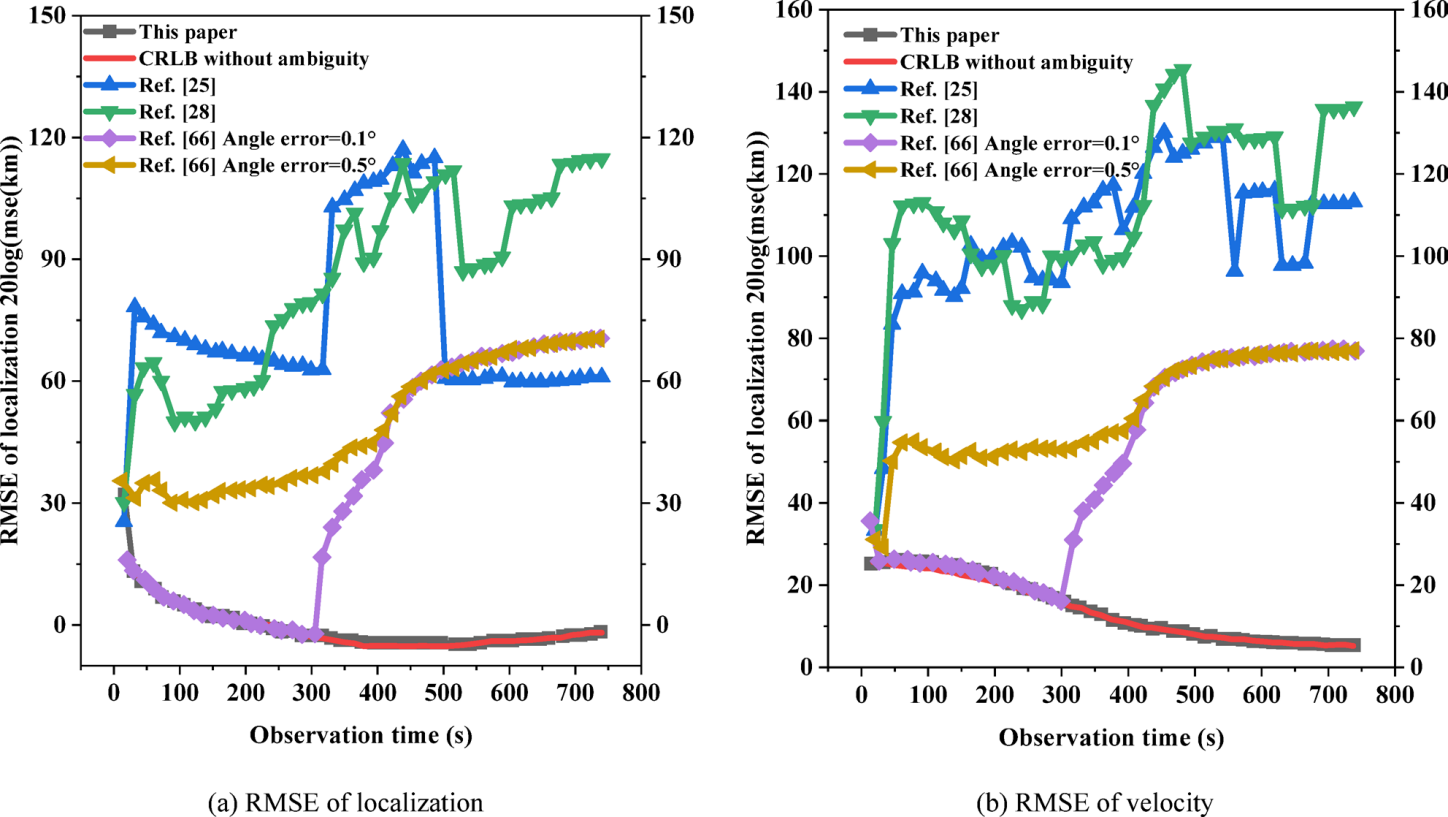
Tracking scenario for low-speed targets.

**Fig. 17:**
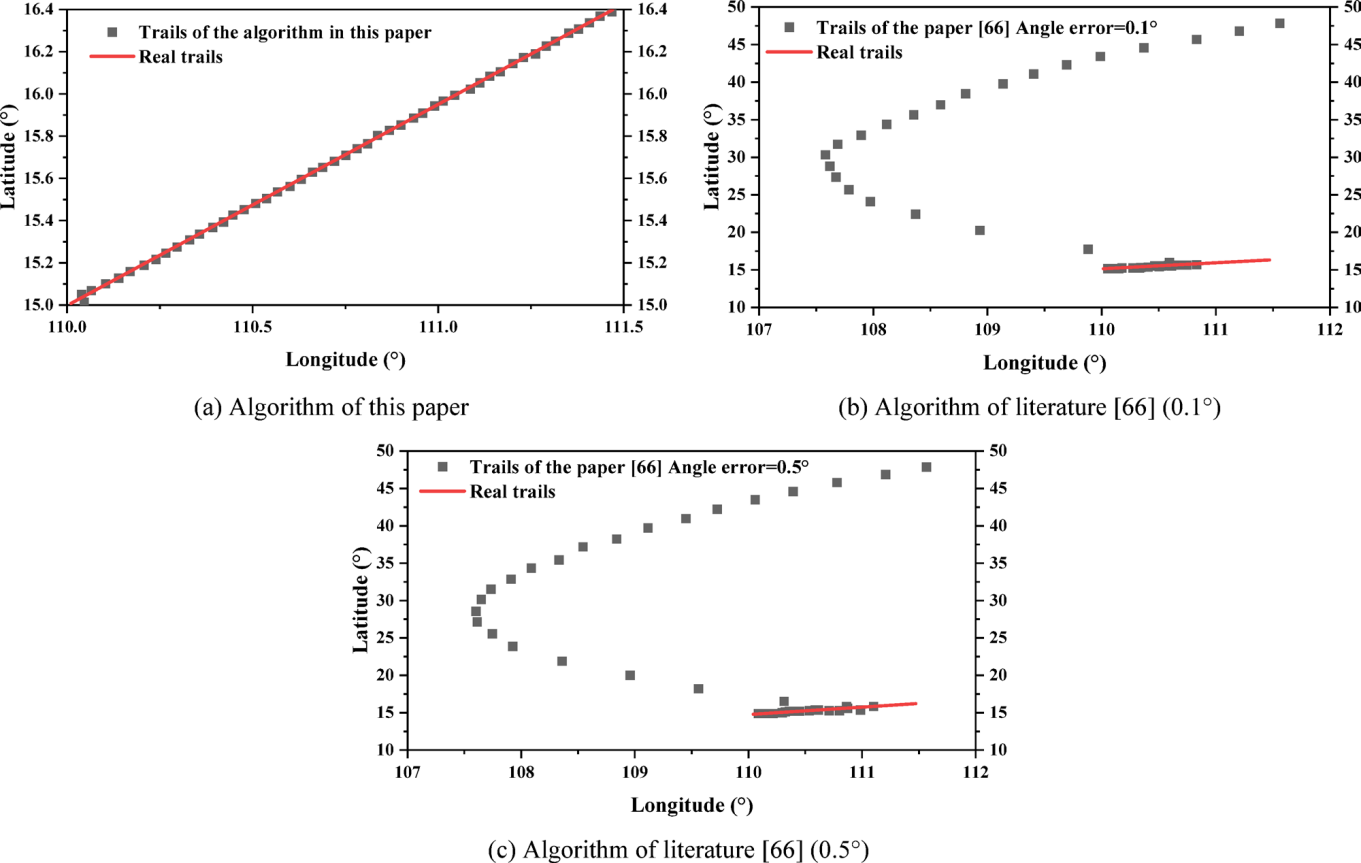
Tracking scenario for high-speed targets.

**Fig. 18:**
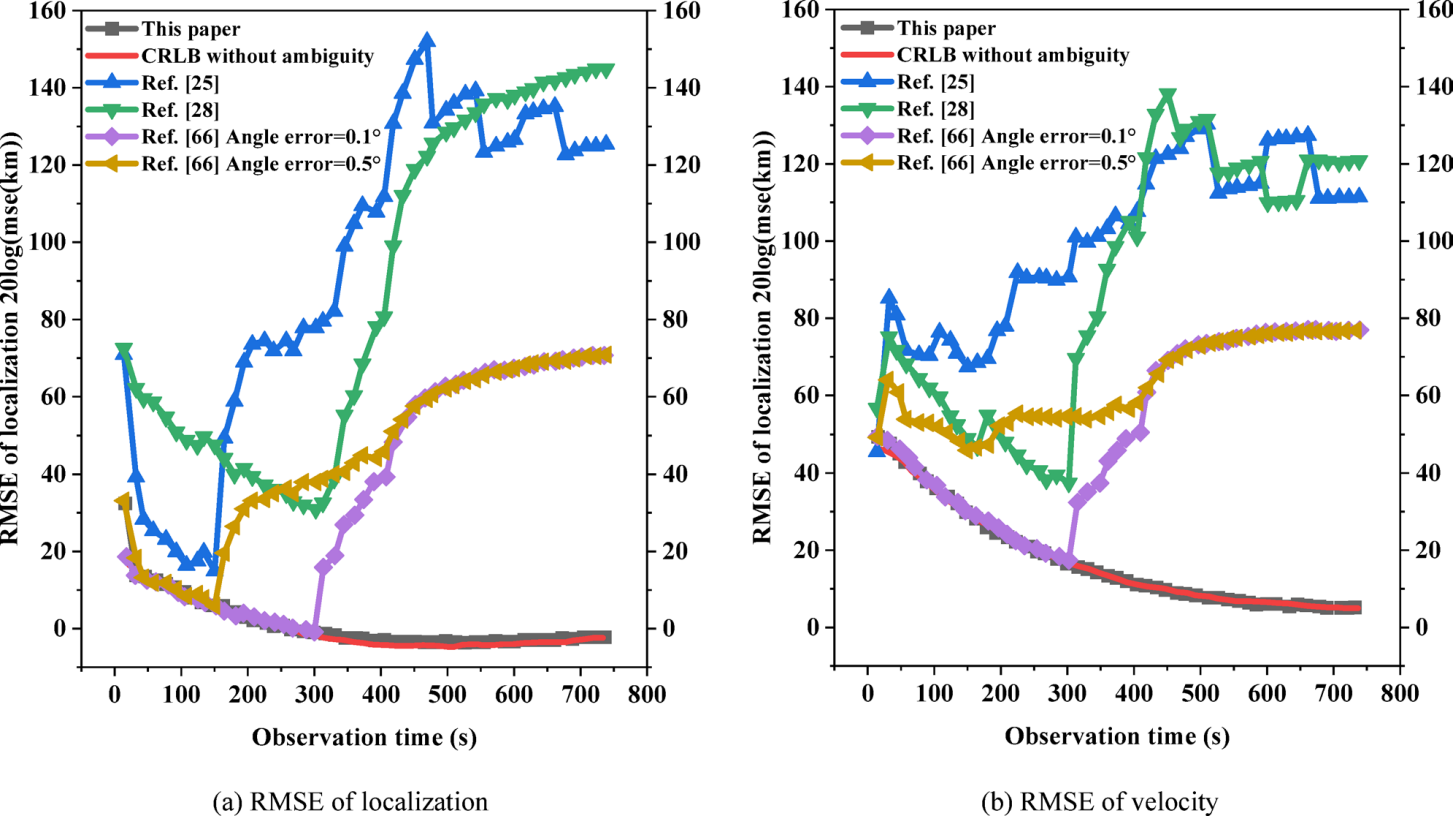
Tracking performance for high-speed targets.

In the above equation, *u* and *v* are the real localization and velocity of the radar emitter, respectively; ${\hat {u}_m}$ and ${\widehat {v}_m}$ are the estimated values of the *m*-th Monte Carlo experiment; *M* = 200. The tracking scenario and performance comparison are shown in Figs. 15 and 16. The high-speed targets does isometric cruising motion at 300 m/s, and *V*_max_ = 300 m/s. The rest of the experimental parameters are the same as the low-speed targets, and the tracking scenario and performance comparison are shown in Figs. 17 and 18.

From the analysis of Figs. 15 and 17, the tracking trails of the algorithm in this paper basically matches the real trails of the target as the observation time increases, regardless of the low-speed targets or the high-speed targets. The computation times of literature^25,28,66^, and this study were 173.8ms, 96.4ms, 133.5ms, and 118.6ms, respectively. Although the computation time of this study is not the shortest, it still has a significant competitive advantage and the highest accuracy. The two deep learning based methods in literature^25,28^ both exhibit significant deviations along the entire trajectory. Both cases of the algorithm in the literature^66^ diverge at the late stage of tracking, where the algorithm in the literature^66^ completely deviates from the real trails from the beginning in the low-speed scenario when the angle error is 0.5°. From Figs. 16 and 18, with the increase of observation time, the performance of the algorithm in this paper gradually reaches the BCRLB without ambiguity. The computation times of literature^25,28,66^, and this study were 216.5ms, 142.8ms, 152.3ms, and 134.4ms, respectively. This study not only had the shortest computation time, but also had the highest accuracy. The performance of the algorithm in the literature^66^ can reach the BCRLB at the beginning with the angle error of 0.1°, while the performance starts to deviate from the BCRLB as the observation time increases; when the angle error increases to 0.5°, the filtering results diverge even earlier. It can be seen that the algorithm of the literature^66^ is combined with direction finding assistance for ambiguity resolution for time-difference, which is influenced by the directional accuracy, which is particularly significant for low-speed targets. The ambiguity resolution for time-difference in this scenario is only possible with extremely small angle errors. Without imposing any auxiliary methods, the algorithm in this paper can effectively achieve the ambiguity resolution for time-difference for the radar emitter of HPRF in cruising motion with only measurements for time-difference from three satellites.

## Conclusion

In order to solve the problems of low localization accuracy for time-difference fusion of multiple satellites and ambiguity in localization and tracking, the localization algorithm and ambiguity resolution algorithm for time-difference fusion of multiple satellites are thoroughly studied.

1. The localization algorithm for time-difference fusion of three satellites based on Gaussian-Newton iteration is proposed. The time-difference equations observed during satellite passes are combined with elevation observation equations to construct an overdetermined cost function, which is then solved using Gaussian-Newton iteration to address the nonlinear least squares problem. Experimentally, it is concluded that the algorithm in this paper outperforms other advanced localization methods and achieves the CRLB of fusion localization.
2. The ambiguity resolution algorithm for localization and tracking with time-difference fusion based on the Kalman filtering combined with the Gaussian mixture model is proposed. The mathematical model of ambiguity for time-difference is established, the calculation method of time-difference window and the number of ambiguous time-difference is given, and the measurements of ambiguous time-difference are approximated by Gaussian mixture model. For stationary targets, the ambiguity resolution algorithm based on Kalman filtering combined with Gaussian mixture model (KFGMM) for localization and tracking is proposed; for cruising targets, the ambiguity resolution algorithm based on capacitive Kalman filtering combined with Gaussian mixture model (CKFGMM) for localization and tracking is proposed. Experiments show that with the increase of filtering time, the algorithm in this paper can achieve BCRLB and outperforms the algorithm combining with direction finding assistance for ambiguity resolution for time-difference.

In future research, it is necessary to study the localization of multiple radar emitters. The signals from multiple emitters may interfere with each other, making signal separation difficult and significantly increasing computational complexity. At the same time, the ambiguity of time differences between multiple radar emitters will become more complex and require more advanced algorithms to solve. Therefore, in the future, advanced signal processing techniques such as adaptive filtering, blind source separation, etc. can be used to separate and identify signals from different radar emitters; combining multiple models (such as Gaussian mixture model, particle filter, etc.) to process signals from multiple emitters, improving localization accuracy and robustness. This study currently only focuses on precise simulation and does not involve the use of real data or the analysis of the impact of environmental and atmospheric noise. In practical applications, environmental and atmospheric noise can significantly affect the accuracy of positioning algorithms, and this factor deserves further exploration in the future.

## Data Availability

The data that support the findings are available from corresponding author upon reasonable request.
